# PD-L1 expression on circulating tumor cells and platelets in patients with metastatic breast cancer

**DOI:** 10.1371/journal.pone.0260124

**Published:** 2021-11-15

**Authors:** Elizabeth P. Darga, Emily M. Dolce, Fang Fang, Kelley M. Kidwell, Christina L. Gersch, Steven Kregel, Dafydd G. Thomas, Anoop Gill, Martha E. Brown, Steven Gross, Mark Connelly, Michael Holinstat, Erin F. Cobain, James M. Rae, Daniel F. Hayes, Costanza Paoletti

**Affiliations:** 1 Breast Oncology Program, Department of Internal Medicine, University of Michigan Medical School, Ann Arbor, Michigan, United States of America; 2 University of Michigan Rogel Cancer Center, University of Michigan, Ann Arbor, Michigan, United States of America; 3 Department of Biostatistics, School of Public Health, University of Michigan, Ann Arbor, Michigan, United States of America; 4 Michigan Center for Translational Pathology, University of Michigan, Ann Arbor, Michigan, United States of America; 5 Menarini Silicon Biosystems, Huntingdon Valley, Pennsylvania, United States of America; 6 Departments of Pharmacology and Internal Medicine, University of Michigan Medical School, Ann Arbor, Michigan, United States of America; Universite de Nantes, FRANCE

## Abstract

**Background:**

Immune checkpoint inhibition is effective in several cancers. Expression of programmed death-ligand 1 (PD-L1) on circulating tumor or immune effector cells could provide insights into selection of patients for immune checkpoint inhibition.

**Methods:**

Whole blood was collected at serial timepoints from metastatic breast cancer patients and healthy donors for circulating tumor cell (CTC) and platelet PD-L1 analysis with a phycoerythrin-labeled anti-human PD-L1 monoclonal antibody (Biolegend clone 29E.2A3) using the CellSearch^®^ assay. CTC PD-L1 was considered positive if detected on at least 1% of the cells; platelet PD-L1 was considered positive if ≥100 platelets per CellSearch frame expressed PD-L1.

**Results:**

A total of 207 specimens from 124 metastatic breast cancer patients were collected. 52/124 (42%) samples at timepoint-1 (at or close to time of progressive disease) had ≥5 CTC/7.5ml whole blood. Of those, 21 (40%) had positive CTC PD-L1. In addition, platelet PD-L1 expression was observed in 35/124 (28%) at timepoint-1. Platelet PD-L1 was not detected in more than 70 specimens from 12 healthy donors. Platelet PD-L1 was associated with ≥5 CTC/7.5ml whole blood (p = 0.0002), less likely in patients with higher red blood cell counts (OR = 0.72, p<0.001) and a history of smoking tobacco (OR = 0.76, p<0.001). Platelet PD-L1 staining was not associated with tumor marker status, recent procedures or treatments, platelet-affecting drugs, or CTC PD-L1 expression.

**Conclusion:**

PD-L1 expression was found in metastatic breast cancer patients on both CTC and platelets in an independent fashion. Inter-patient platelet PD-L1 expression was highly heterogeneous suggesting that it is a biological event associated with cancer in some but not all patients. Taken together, our data suggest that CTC and platelet PD-L1 expression could play a role in predicting which patients should receive immune checkpoint inhibition and as a pharmacodynamics biomarker during treatment.

## Introduction

Immune checkpoint inhibition (ICPi) with antibodies to programmed cell death 1 (PD-1) and its ligand (PD-L1) is effective in several malignancies [1, 2]. PD-L1 expression on tumor or infiltrating immune cells in malignant tissue predicts benefit from anti-PD-L1/PD-1 therapies [3]. Other predictors of response to ICPi include presence of tumor infiltrating lymphocytes, human leukocyte antigen (HLA) status, high tumor mutation burden or surrogates of it, antigen presenting cells, and the host microbiome [4–6].

PD-L1 expression is dynamic. Tissues tested for PD-L1 are often collected at time periods long before the patient is treated with ICPi therapy [3]. Evaluation of circulating tumor biomarkers in blood, designated “liquid biopsies” may provide real-time estimates of tumor status [7]. In this regard, elevated circulating tumor cell (CTC) enumeration is prognostic in several metastatic epithelial cancers [8–11]. PD-L1 expression of CTC might provide additional information regarding potential clinical benefit from anti-PD-L1/PD-1 therapy [12–19].

In the process of evaluating PD-L1 expression on CTC with the CellSearch^®^ CTC detection system [Menarini Silicon Biosystems (MSB)], we also observed PD-L1 staining of platelets. Therefore, we conducted a prospective preliminary study to determine the incidence of these findings in patients with metastatic breast cancer (MBC). We hypothesize both CTC and platelet expression of PD-L1 might be another mechanism of immune-checkpoint modulation, serving as additional predictive and monitoring factors for ICPi therapy.

## Methods

### Patient accrual and characteristics

Female patients with MBC of any subtype were enrolled into this prospective single-institution pilot study. Initial blood draws were at or close to the time the patient progressed on her most recent therapy. Serial blood draws were not at clinically relevant timepoints, but rather for additional experimentation and observation. Control blood was collected from male and female healthy donors. All subjects signed written informed consent approved by the Internal Review Board at the University of Michigan in accordance with the Declaration of Helsinki. Clinical characteristics were obtained by chart review and abstraction.

### Blood collection, processing, CTC enumeration, and phenotyping

Whole blood (WB) was collected into 10ml CellSave tubes (MSB, Huntingdon Valley, PA) and processed through the CellSearch^®^ (CXC kit) system (MSB, Huntingdon Valley, PA) as previously described [13, 20]. PD-L1 staining was performed using a phycoerythrin-labeled anti-human PD-L1 monoclonal antibody (Biolegend clone 29E.2A3; Cat# 329705, RRID:AB_940366; Biolegend, San Diego, CA) at 3.5 ug/ml, using methods similar to those previously described for other phenotypic markers [13, 14, 20, 21].

### Quantifying platelets from CellSearch^®^ enriched product

The contents from CellSearch cartridges were extracted using gel loading tips coated in 2% BSA/PBS and placed into Eppendorf tubes. The number of platelets/ul was determined using the Hemavet^®^ hv950 (Drew Scientific, Miami Lakes, FL).

### Cell culture for *in vitro* experiments

Human breast cancer cell lines were cultured in a sterile incubator at 37°C with 5% CO_2_. Characteristics for each cell line are described in **S1 Table**. Expression status of ER, PgR, HER2, and PD-L1 for each cell line were previously described by Mittendorf et al. [22] (**S1 Table**). The cell lines used for *in vitro* experiments were used within two passages of defrosting. All cell lines were routinely tested for mycoplasma contamination and identified by Short Tandum Repeat profiling as previously described [20].

### Platelet characterization of CellSearch^®^ enriched product

Contents from CellSearch^®^ cartridges were extracted, incubated for 30 minutes on ice with APC/Cy7 anti-human CD-42b and CD-41 monoclonal antibodies directed against platelets (clone HIP1 RRID: AB_2616778 Cat# 303920 and HIP8 RRID: AB_10896432 Cat#303715, respectively; Biolegend, San Diego, CA), centrifuged at 800g for 20 minutes, washed twice with 1% BSA/PBS, centrifuged for 8 minutes at 94g onto poly-lysine coated slides, then examined by fluorescence microscopy at 200X for platelet and PD-L1 expression.

### Platelet isolation from whole blood

Platelet rich plasma (PRP) was isolated from 7.5ml WB collected in CellSave tubes by 200g centrifugation for 10 minutes at room temperature (RT). Platelets were isolated by a second spin of 2,000g at RT and the platelet poor plasma (PPP) was centrifuged two additional times at 2,000g to remove residual platelets. The PPP and platelet pellet were each resuspended with dilution buffer to a total volume of 14ml. The sample tubes were taped to the 4ml mark to simulate the red blood cell layer, enabling the automated CellSearch^®^ to process the sample. Because these samples lacked whole cells necessary to focus the CellTracks Analyzer camera, DAPI coated magnetic beads provided by MSB were added to each cartridge to permit scanning.

### PD-L1 knockdown in cultured human breast cancer cells

Silencer^®^ select pre-designed siRNA (s26547, s26548, s26549; Life Technologies, Carlsbad, CA) was used to knockdown PD-L1 expression in MDA-MB-231 cells. Two nonsense controls were used separately, one from Dharmacon and one from Silencer^®^ select. The cells were transfected using Lipofectamine RNAiMax reagent (Invitrogen, Carlsbad, CA) in Opti-MEM medium with a final concentration of 25 pmol siRNA per well following manufacturer’s instructions (**S1 File**).

### Western blot analyses

PD-L1 protein expression in cultured human breast cancer cells was confirmed using Western blot analyses. For each sample, 35 ug of protein lysate was loaded onto 4-12% acrylamide gels. PVDF membrane was blocked with 5% milk/TBS-T and incubated with anti-PD-L1 rabbit primary monoclonal antibody (E1L3N Rabbit mAb; Cat# 13684, RRID:AB_2687655, Cell Signaling Technologies, Danvers, MA) and subsequently with horseradish peroxidase-linked anti-rabbit IgG secondary mAb (Cat# 7074, RRID:AB_2099233, Cell Signaling Technologies, Danvers, MA). Horseradish peroxidase linked β-actin was used as a loading control (8H10D10, Cat# 3700, RRID:AB_2242334, Cell Signaling Technologies, Danvers, MA).

### PD-L1 gene expression

Cell lysates were prepared using trizol and homogenized using a 23 gauge sterile syringe. RNA was extracted using the Qiagen mini-RNA extraction kit (Qiagen, Hilden, Germany) following manufacturer’s instructions and included an Ambion DNA clean up kit (Invitrogen, Carlsbad, CA). Purified RNA was interrogated with the Promega Reverse Transcription Kit for RT-PCR (Promega, Madison, WI). Template cDNA was further amplified using sybergreen PCR with primer pairs designed for human PD-L1 (Integrated DNA Technologies, Coralville, IA). Samples were analyzed in triplicate using a CFX96 Real-Time PCR detection system (Bio-Rad, Hercules, CA). All samples were normalized to GAPDH as previously described [23] and measured as fold change of PD-L1 expression from untreated MDA-MB-231 cells. All primers were diluted to a working concentration of 250nM. PD-L1 primer pair sequences are as follows: primer pair 1 5’CTTCCGTTTAAGAAAAGGGAGAA3’/ 5’TTACGTCTCCTCCAAATGTGT3’; primer pair 2 5’CTGACATTCATCTTCCGTTTAAG3’/5’CGTCTCCTCCAAATGTGTATCA3’; primer pair 3 5’GACATTCATCTTCCGTTTAAGAAA3’/5’CGTCTCCTCCAAATGTGTATCA3’.

### Statistical analysis

The distribution of CTC PD-L1 and platelet PD-L1 expression in all blood samples was summarized using descriptive statistics. A marginal model with a logit link using generalized estimating equations (GEE) was fit to explore whether platelet PD-L1 positivity varied across the timepoints. To evaluate the association between timepoint-1 PD-L1 expression with timepoint-1 CTC and platelets, we performed a Pearson’s chi-squared test and Fisher’s exact test, respectively, each with significance level p = 0.05.

Marginal models estimated using GEE assuming an independent working correlation structure were used to explore the clinical/pathological features associated with CTC and platelet PD-L1 expression. For CTC PD-L1 expression, the outcome consisted of the count of PD-L1 positive CTC using samples with >0 CTC/7.5ml WB. Platelet PD-L1 expression was dichotomized between negative (<100 PD-L1 positive platelets per frame) or positive (≥100 PD-L1 positive platelets per frame) expression. Associations between features of interest with PD-L1 expression were assessed singly. The false discovery rate approach was used to adjust the type I error to claim statistical significance with p-value ≤0.001.

Data management and analysis were conducted using Statistical Analysis System (SAS) statistical software, version 9.4 (Statistical Analysis System, RRID:SCR_008567, SAS Institute Inc., Cary, NC, USA).

## Results

### CTC PD-L1 expression in patients with MBC at timepoint-1

Blood samples from 124 patients with MBC were assessed for CTC PD-L1 expression at timepoint-1 and subsequent timepoints in selected cases (**S1 Fig**). Timepoint-1 was at or close to any time that a patient was found to have progressive disease.

**Fig 1:**
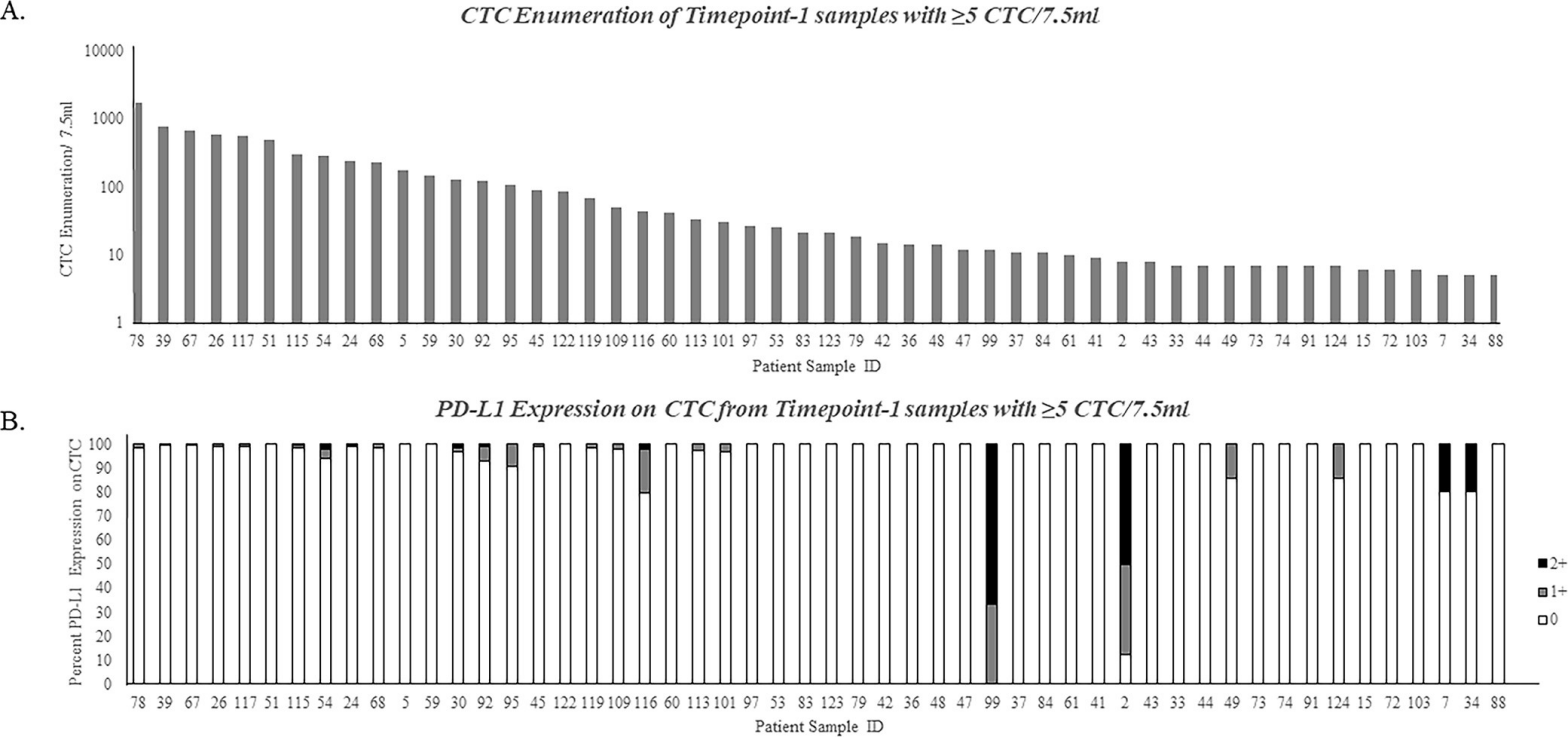
CTC Enumeration and CTC PD-L1 Expression at timepoint-1. Whole blood (WB) specimens from 124 patients with metastatic breast cancer were processed using the CellSearch^®^ system. Circulating tumor cell (CTC) enumeration and PD-L1 expression are displayed for the 52 (42%) subjects who had elevated CTC (≥5 CTC/7.5ml WB) at timepoint-1. Numbers under each bar represent subject study designations. A. Distribution of CTC enumeration. Each bar represents the number of CTC in an individual patient sample. B. Distribution of CTC PD-L1 staining. Colors represent the percent of CTC that expressed PD-L1, on a scale of 0-3+ (see Methods for details). 0 (■ white), 1+ (■ gray), and 2+ (■ black). No patients had 3+ staining for CTC PD-L1.

Of the 124 samples at timepoint-1, 52 (42%) had elevated CTC (≥5/7.5ml WB) (**Table 1**). Twenty-one (40%) of these 52 specimens had ≥1% CTC PD-L1 expression of 1-2+ [median 15.2% (range 1-100%); **Fig 1; Table 1**], within a semi-quantitative grading system we developed (**S2 Fig**). Although phenotyping data is typically reported only for patients with ≥5/7.5ml WB, for patients in this study with 1-4 CTC/7.5ml WB at least one PD-L1 positive CTC was also observed in 9/30 (30%) patients (**Table 1**).

**Table 1:** CTC PD-L1 expression at timepoint-1.

#CTC/7.5ml WB	CTC Enumeration^a^	PD-L1 Positive^b^
0 CTC	42	N/A
1-4 CTC	30	9 (30%)
≥5 CTC	52	21 (40%)
**Total**	**124**	**30 (24%)**

^a^ Number of patients assessed for CTC/7.5ml WB at timepoint-1.

^b^ Number (%) of patients with ≥1% CTC PD-L1 expression at timepoint-1.

### Platelet PD-L1 expression in patients with MBC

In the classic CellSearch^®^ system, leukocytes are identified by staining with fluoresceinated anti-CD45, and platelets are not visualized. During CTC PD-L1 expression analysis using CellSearch^®^, we observed PD-L1 staining on what visually appeared to be platelets. In extensive evaluations of WB using CellSearch^®^ standard antibodies, as well as investigational studies using labeled antibodies against a number of other biomarkers, we have not observed platelet staining [13, 14, 20, 21]. Platelet PD-L1 was not observed in over 70 WB samples collected longitudinally from 12 healthy donors spiked with MDA-MB-231 cells and processed through CellSearch^®^ and stained with the 29E.2A3 PD-L1 antibody. Therefore, we further investigated this curious finding.

In the CellSearch^®^ system, a platelet specific marker cannot be used simultaneously with anti-PD-L1, since only one additional florescence channel is available for phenotyping. Therefore, using cytospins, we demonstrated that the PD-L1 positive, non-nucleated objects observed in CellSearch^®^ co-stained with anti-platelet antibodies, confirming that they were indeed, platelets (**S3 Fig**). Platelet PD-L1 staining was not an artifact of the CellSave tube fixative (**S4 Fig** and **S2 Table**). When PD-L1 positive platelets were visible, they were evenly distributed throughout the CellSearch^®^ cartridge as is visualized in images of three different frames within one CellSearch patient cartridge (**S5 Fig**). Additionally, we tested the specificity of the PD-L1 antibody we employed, 29E.2A3, by siRNA knockdown and cell line staining (**S6** and **S7 Figs**).

The CellSearch^®^ system is designed to enrich for cells expressing the epithelial cell adhesion molecule (EpCAM). However, CellSearch^®^ does not enrich epithelial cells to purification, and some leukocytes, which are EpCAM negative and CD-45 positive, are routinely carried-over during the enrichment process in all samples. Hence, the addition of fluoresceinated anti-CD45 in the CellSearch^®^ system in order to distinguish the two. However, it was not previously recognized that platelets, as well, are carried over during the enrichment process, and since platelets would not mimic CTC, no added staining is included in the CellSearch^®^ system. Therefore, we tested the carry-over of platelets in the CellSearch® assay.

**Fig 2:**
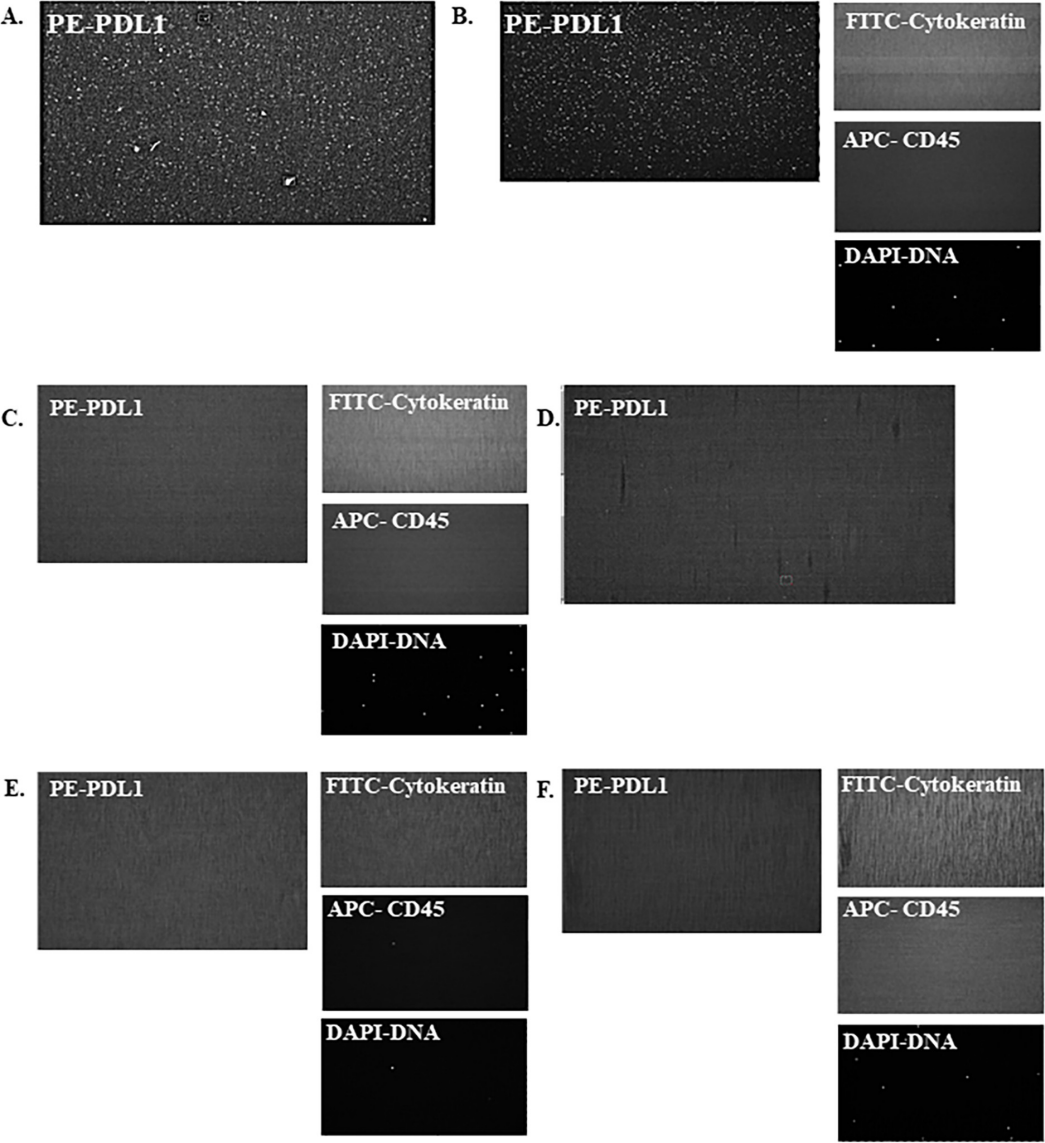
PD-L1 expression on platelets. Whole Blood, a pure platelet pellet, and platelet poor plasma from two patients, one with positive platelet PD-L1 (A) and one with negative platelet PD-L1 (D), was processed through CellSearch® in parallel with PD-L1 staining in the 4th channel. A. Fluorescent image of positive platelet PD-L1 (PE conjugated) expression obtained from initial whole blood processed through CellSearch® using the classic method. B. Fluorescent images of PD-L1 (PE conjugated), cytokeratin (FITC-conjugated), CD-45 (APC conjugated), and DNA (DAPI) for platelet pellet from patient in panel A with positive platelet PD-L1 processed through CellSearch®. C. Fluorescent images of PD-L1 (PE conjugated), cytokeratin (FITC-conjugated), CD-45 (APC conjugated), and DNA (DAPI) for platelet poor plasma from patient in panel A with positive platelet PD-L1 processed through CellSearch®. See Methods for details. D. Florescent image of negative platelet PD-L1 (PE conjugated) expression obtained from initial whole blood processed through CellSearch® using the classic method. E. Florescent images of PD-L1 (PE conjugated), cytokeratin (FITC-conjugated), CD-45 (APC conjugated), and DNA (DAPI) for platelet pellet from patient in panel D with negative platelet PD-L1 processed through CellSearch®. F. Florescent images of PD-L1 (PE conjugated), cytokeratin (FITC-conjugated), CD-45 (APC conjugated), and DNA (DAPI) for platelet poor plasma processed from patient in panel D with negative platelet PD-L1 through CellSearch®.

For patient samples in which platelet PD-L1 staining was observed using the classic CellSearch^®^ method (**Fig 2A**), PD-L1 staining was maintained in the aliquot containing only the platelet pellet (**Fig 2B**). Staining for CD-45 (APC) and CK (FITC) in this aliquot was negative, demonstrating that the sample was clear of white blood cell (WBC) and CTC carryover (**Fig 2B**). Platelet poor plasma (PPP) did not have PD-L1 staining, confirming that the PD-L1 staining was on platelets (**Fig 2C**). In both platelet pellet and PPP samples, the DAPI fluorescence was seen only on DAPI coated magnetic beads (**Fig 2B–2E**) added to permit scanning of the samples. For patient samples that did not have platelet PD-L1 staining present in the classic CellSearch^®^ method, neither the platelet pellet nor PPP displayed PD-L1 staining (**Fig 2D–2F**). Taken together, these data confirmed that the non-CTC PD-L1 staining is on platelets and that platelets do indeed carry-over during the CellSearch enrichment process.

In a subset of patients, the number of platelets carried-over into the CellSearch cartridge was quantified using a Hemavet. Platelet PD-L1 staining was independent of number of platelets within the CellSearch^®^ cartridge as well as routine clinical complete blood count determined on the same day as the research blood collection (**S3 Table**).

Inter-patient platelet PD-L1 expression was heterogeneous. Using a semi-quantitative scale (**S8 Fig**), 41 (33%), 48 (39%), 24 (19%), and 11 (9%) of 124 samples at timepoint-1, had 0, <100, 100-1,000, and >1,000 PD-L1 positive platelets/frame of the CellSearch^®^ cartridge, respectively (**Table 2**). Using an arbitrarily designated cutoff of ≥100 PD-L1 positive platelets/frame as positive, 35 (28%) samples were positive for platelet PD-L1 expression at timepoint-1.

**Table 2:** Platelet PD-L1 distribution in all samples.

Platelet PD-L1 Score^a^	Blood Draw Timepoints				
	1	2	3	4	5
	N (%)	N (%)	N (%)	N (%)	N (%)
	124	59	16	6	2
**Negative** ^**b**^	**89 (72%)**	**39 (66%)**	**9 (56%)**	**2 (33%)**	**0**
0	41 (33%)	16 (27%)	5 (31%)	0	0
<100	48 (39%)	23 (39%)	4 (25%)	2 (33%)	0
**Positive** ^**b**^	**35 (28%)**	**20 (34%)**	**7 (44%)**	**4 (67%)**	**2 (100%)**
100-1,000	24 (19%)	12 (20%)	5 (31%)	2 (33%)	0
>1,000	11 (9%)	8 (14%)	2 (13%)	2 (33%)	2 (100%)

^a^Average platelet count/3 CellSearch Frames (see **S1 File** for details).

^b^Arbitrary classification of platelet PD-L1 staining (see **S1 File** for details).

N = number.

At timepoint-1, platelet PD-L1 expression was associated with elevated CTC levels, but not with CTC PD-L1 expression. Twenty-four of the 52 (46%) samples with ≥5 CTC/7.5ml WB, but only 11/72 (15%) samples with <5 CTC/7.5ml WB had PD-L1 positive platelets (p = 0.0002) (**Table 3**). Platelet PD-L1 expression was independent of CTC PD-L1 expression for both samples with ≥5 CTC/7.5ml WB (p = 0.99) and <5 CTC/7.5ml WB (p = 0.34) (**Table 3**).

**Table 3:** Association of platelet PD-L1 score and CTC enumeration and PD-L1 expression at timepoint-1.

		Association of Platelet PD-L1 Positivity with:										
		CTC Enumeration (≥5/7.5ml WB)^c^			CTC PD-L1 Expression ^d^^,^ ^e^							
		N^0^ of CTC/7.5 ml WB				N^0^ of CTC/7.5 ml WB						
						0	1-4			≥5		
						N	N			N		
						(%)	(%)			(%)		
		(0-4)	(≥5)	Total	*CTC PD-L1 Score* ^*b*^	NA	NEG	POS	Total	NEG	POS	Total
		N	N									
		(%)	(%)									
***PlateletPD-L1 score*** ^***a***^	***NEG (0 - <100)***	61 (49%)	28 (23%)	89 (72%)		39 (54%)	15 (21%)	7 (10%)	61 (85%)	15 (29%)	13 (25%)	28 (54%)
	***POS (100 - >1*,*000)***	11 (9%)	24 (19%)	35 (28%)		3 (4%)	6 (8%)	2 (3%)	11 (15%)	16 (31%)	8 (15%)	24 (46%)
	***Total***	72 (58%)	52 (42%)	124		42 (58%)	21 (29%)	9 (13%)	72	31 (60%)	21 (40%)	52

^a^Arbitrary classification of platelet PD-L1 staining based on average platelet count/3 CellSearch Frames (see **S1 File** for details).

^b^Number (%) of patients with ≥5 CTC/7.5ml WB enumeration and <1% (NEG) or ≥1% (POS) CTC PD-L1 expression.

^c^Chi-squared test for comparison Platelet PD-L1 NEG vs. POS according to CTC PD-L1 enumeration; p-value = 0.0002.

^d^Chi-squared test comparison Platelet PD-L1 NEG vs. POS according to CTC PD-L1 expression if 0-4 CTC/7.5 ml whole blood; p-value = 0.34.

^e^Fisher’s exact test comparison Platelet PD-L1 NEG vs. POS according to CTC PD-L1 expression if CTC ≥5 p-value = 0.99.

### Association of CTC PD-L1 and platelet PD-L1 expression with clinical/pathological features

We assessed associations of CTC PD-L1 and platelet PD-L1 expression with clinical and pathological features (**S4–S8 Tables**).

#### CTC PD-L1 and clinical/pathological features

Only patients (n = 91) with ≥1 CTC/7.5ml WB at at least one timepoint were included in the analysis of association of CTC PD-L1 and clinical/pathological features. By univariable analysis, CTC PD-L1 positivity was less likely in patients with ER+ compared to triple negative MBC at either the time of 1^st^ clinical metastatic biopsy (RR = 0.33, p<0.001) and at a subsequent, later metastatic biopsy (RR = 0.31, p<0.001). Similarly, CTC PD-L1 was less likely in patients with HER2+ compared to triple negative MBC at the time of either metastatic biopsy (RR = 0.22, p<0.001 for both) (**S5 Table**). CTC PD-L1 was also less likely in patients with bone only disease compared to patients without bone disease (RR = 0.14, p<0.001). CTC PD-L1 was significantly increased in patients currently receiving or who had just progressed on either endocrine therapy (ET) (RR = 3.19, p<0.001) or CDK4/6 inhibitors (RR = 4.11, p<0.001) (**S5 Table**). CTC PD-L1 was not associated with anticoagulant drugs, although only 13 patients were on a dedicated anticoagulant medication (rivaroxaban, enoxaparin, apixaban, clopidogrel) (**S5 Table**).

In multivariable analysis, in contrast to the univariable analysis, CTC PD-L1 was significantly higher in patients with ER+ compared to triple negative disease (RR = 2.56, p = 0.007) and HER2+ compared to triple negative disease (RR = 3.14, p = 0.042). In concert with the univariable analysis, it was associated with prior treatment or progression on CDK4/6 inhibitors (RR = 3.60, p = 0.008) (**Table 4**). Likewise, CTC PD-L1 was significantly lower in patients with bone only disease (RR = 0.09, p<0.001) or with bone and other sites of disease (RR = 0.19, p<0.001) **(Table 4**).

**Table 4:** Multivariable results of factors of interest with CTC PD-L1 positive rate ^a^ and Platelet PD-L1 positivity ^b^.

Characteristics	Category	Rate Ratio^c^ or Odds Ratio^d^ of PD-L1 Positivity (95% CI)	P-value ^e^
**CTC PD-L1**			
Most recent metastatic hormone receptor status^f^	(Overall)		0.03
	ER+, HER2- vs. Triple Neg	2.56 (1.298, 5.058)	0.007
	HER2+ vs. Triple Neg	3.14 (1.040, 9.492)	0.042
Disease Site	(Overall)		<0.001
	Bone + other site vs. Other site (no bone)	0.19 (0.078, 0.462)	<0.001
	Bone only vs. Other site (no bone)	0.09 (0.034, 0.243)	<0.001
CDK4/6 inhibitor	Yes vs. No	3.60 (1.403, 9.238)	0.008
**Platelet PD-L1**			
RBC (M/ul)	Continuous variable	0.73 (0.642, 0.820)	<0.001

^a^ Only significant factors included in this table. See **S6 Table** for full multivariable analysis.

^b^ Only significant factors included in this table. See **S8 Table** for full multivariable analysis.

^c^ Rate ratio is calculated using Poisson GEE model assuming an independent correlation structure to explore the association between CTC PD-L1 and factors of interest.

^d^ Platelet PD-L1 positivity is binary positive (≥100 PD-L1 positive platelets) or negative (<100 PD-L1 positive platelets) by CellSearch, odds ratio is calculated using GEE model assuming an independent correlation structure to explore the association between Platelet PD-L1 and factors of interest.

^e^ Statistical significance is any p<0.05.

^f^ The metastatic biopsy that was performed closest to the time the blood specimen for this study was collected.

#### Platelet PD-L1 and clinical/pathological features

By univariable analysis, platelet PD-L1 positivity was higher in patients who had increased numbers of CTC (OR = 1.03 for each 100 CTC/7.5ml WB increase, p<0.001) and in patients with ≥5 vs. <5 CTC (OR = 1.45, p<0.001), but as noted above was independent of CTC PD-L1 status (**S7 Table**). It was significantly lower in patients with increased number of red blood cell counts (univariable OR = 0.72 for each M/ul increase, p<0.001; multivariable OR = 0.73, p<0.001) (**S7 Table**, **Table 4**). Platelet PD-L1 was also statistically lower in patients who were current vs. passive/never smokers by univariable analysis (OR = 0.76, p<0.001)(**S7 Table**). Anticoagulant drugs did not appear to affect platelet PD-L1 expression. However, since only a single patient was on clopidogrel and NSAID or aspirin use was taken on an as needed basis and often not recorded, no association with specific platelet-affecting agents could be drawn. Platelet PD-L1 expression was not associated with any other identifiable pathological or clinical features (**S7** and **S8 Tables**).

No attempt to associate either CTC or platelet PD-L1 expression and outcomes was performed, since patients were enrolled based on convenience and represented a vast heterogeneity of breast cancer subtypes, treatments, lines of therapy, and follow-up.

### Serial specimen CTC PD-L1 and platelet PD-L1 expression

#### Serial CTC PD-L1

**Fig 3:**
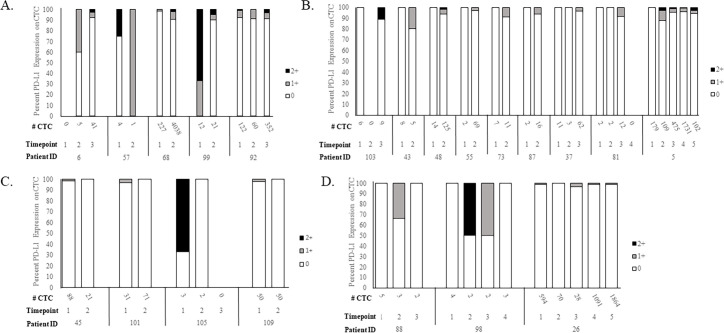
CTC PD-L1 expression at serial timepoints. Whole blood from serial specimens from the same patients was processed through CellSearch® with PD-L1 staining in the 4th channel. Each stacked bar represents distribution of CTC PD-L1 staining as described in Fig 1. Patients in each category are separated by a vertical line. A. Patients (N = 5) that maintained CTC PD-L1 positivity from timepoint-1 to subsequent timepoints. B. Patients (N = 9) that converted CTC PD-L1 negative to positive from timepoint-1 to subsequent timepoints. C. Patients (N = 4) that converted CTC PD-L1 positive to negative from timepoint-1 to subsequent timepoints. D. Patients (N = 3) that had fluctuating CTC PD-L1 expression among multiple timepoints.

Of the 124 patients enrolled, 59 had specimens assessed for CTC PD-L1 and platelet PD-L1 at multiple subsequent timepoints, ranging from 1.5 weeks to 27 months after timepoint-1. Additional information regarding serial sampling such as time between subsequent timepoints, whether the blood was drawn when the patient was on treatment or at progression, and treatment regimen is detailed in **S9 Table**. Of these 59, 35 (59%) had ≥1 CTC/7.5ml in two or more subsequent specimens and 14/35 (40%) were CTC PD-L1 negative at all timepoints. Five (14%) patients maintained CTC PD-L1 positivity (**Fig 3A**), 9 (26%) patients converted CTC PD-L1 status from negative to positive (**Fig 3B**), 4 (11%) patients converted from positive to negative (**Fig 3C**), and 3 (9%) patients had CTC PD-L1 expression fluctuating from negative to positive to negative again at subsequent timepoints (**Fig 3D**).

#### Serial Platelet PD-L1

**Fig 4:**
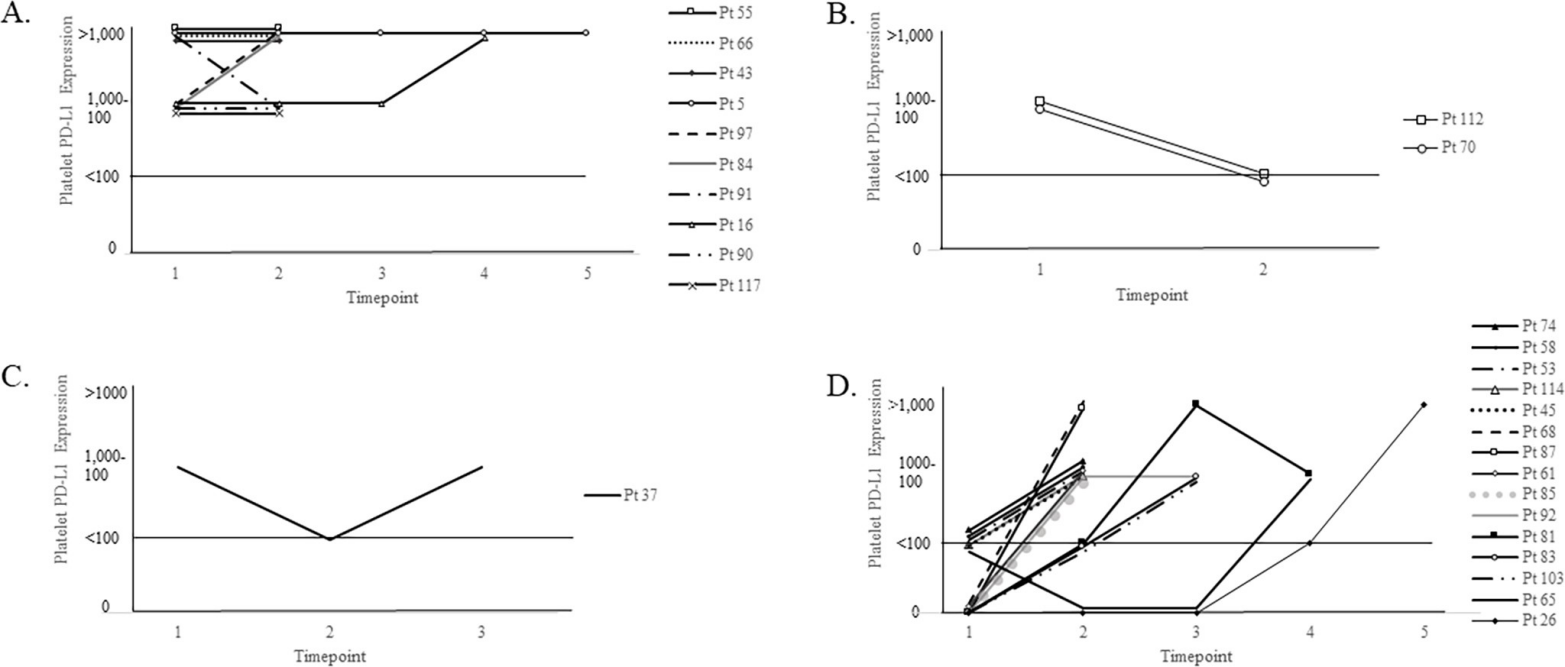
Platelet PD-L1 expression at serial timepoints. Whole blood from serial specimens from the same patients was processed through CellSearch® with PD-L1 staining in the 4th channel. Each line represents platelet PD-L1 staining from separate patients. Any data point that falls at or below the solid black line is considered platelet PD-L1 negative (<100 PD-L1 positive platelets). A. Patients (N = 10) that maintained platelet PD-L1 positive from timepoint-1 to subsequent timepoints. B. Patients (N = 2) that converted from having platelet PD-L1 positive to platelet PD-L1 negative at subsequent timepoints. C. Patient (N = 1) that had platelet PD-L1 expression fluctuating among multiple timepoints. D. Patients (N = 15) that converted from platelet PD-L1 negative at timepoint-1 to platelet PD-L1 positive at subsequent timepoints.

Platelet PD-L1 positivity varied significantly over time in some but not all patients (p = 0.005). Of the 13 patients who had platelet PD-L1 positive at timepoint-1, 10 (77%) maintained positivity at subsequent blood draws (**Fig 4A**). In contrast, 2 (15%) patients converted from platelet PD-L1 positive to negative (**Fig 4B**) and 1 (7%) patient had platelet PD-L1 status fluctuating from positive to negative to positive again among subsequent timepoints (**Fig 4C**). Of the 46 patients who had platelet PD-L1 negative at timepoint-1, 31 (67%) maintained platelet PD-L1 negativity at subsequent blood draws, whereas 15 (33%) patients converted from having platelet PD-L1 negative to positive at a later timepoint (**Fig 4D**).

## Discussion

In this study, using the CellSearch^®^ assay system, we confirmed PD-L1 expression on CTC at timepoint-1 in 17% of patients with MBC. As expected, 42% of these patients had ≥5 CTC/7.5ml WB [9, 24, 25]. Of these, 40% were CTC PD-L1 positive. These data confirm previous reports of CTC PD-L1 expression and are very similar to the 37% CTC PD-L1 positivity reported by Jacot *et al* [17, 26, 27]. However, we observed a lower percentage of CTC PD-L1 positivity than reported by other investigators, in which both the CTC isolation method and the anti-PD-L1 antibody differed from ours [27].

In multi-variable analyses, CTC PD-L1 expression was less likely to be observed in patients with bone metastases compared to those without bone metastases. By univariable analyses, CTC PD-L1 expression was more likely observed in patients currently receiving or who had recently progressed on ET, especially when given in combination with a CDK4/6 inhibitor, at the time of 1^st^ blood draw. Enigmatically, in multi- but not uni-variable analysis, CTC PD-L1 was more likely to be observed in ER+ and HER2+ versus triple negative MBC. The paradoxical finding of CTC PD-L1 association with ER negativity in uni-variable but with positivity in multi-variable analyses is unexplained, but might suggest that positivity is associated with some other feature, such as multiple prior lines of therapy and/or the cancer’s becoming refractory to ET.

Importantly, we also observed platelet PD-L1 expression in 28% of the patients with MBC. Platelet PD-L1 staining was associated with elevated CTC levels, but was independent of CTC PD-L1 expression. Expression of platelet PD-L1 was highly heterogeneous among different MBC patients. Unlike CTC PD-L1 positivity, it was not associated with many distinct clinical or pathologic features, including tumor hormone receptor or HER2 status, apparent burden or site of disease, progressive or stable disease. Importantly, platelet PD-L1 positivity did not appear to be an artifact of causes that might be expected to activate platelets, such as recent surgery or other procedures within the preceding two months, presence of an intravascular indwelling device (port-a-cath), thrombocytopenia, or treatment. Platelet PD-L1 positivity was lower in patients who were current smokers and in those with increased red blood cell counts. For patients with serial blood draws, platelet PD-L1 expression remained stable in 69% of patients. Of the 18 patients that did have a change in platelet PD-L1, 15 (83%) changed from negative to positive over time.

Platelet PD-L1 expression has been previously described by flow cytometry, using the same PD-L1 antibody we employed [28, 29]. These reports have mostly, if not entirely, been within the context of comprehensive analysis of PD-L1 expression on all hematopoietic and immune-effector cells. Furthermore, the prior reports of CTC PD-L1 expression in patients with MBC have not included observations of platelet expression of PD-L1, [17, 26] raising concerns about the specificity of the individual antibodies used to evaluate this marker. Our extensive investigations demonstrated that a) the antibody (29E.2A3) used in our investigations was specific for PD-L1 and b) the elements that were staining for PD-L1 were clearly platelets (**S1 File**).

Platelets prevent hemorrhage, but have many other activities in normal hemostasis, wound healing, and immune function [30, 31]. We speculate that platelet PD-L1 expression might serve in the normal situation to protect epithelial cells from being innocent bystanders in the early immune response to infection. Further, as epithelial cells progress along the malignant continuum [32], some, but not all, may be associated with platelets expressing PD-L1 and commensurate immune suppression. Thus, platelet PD-L1 expression may be a mechanism of tumor escape from immune elimination [5, 33, 34]. More recently, Zaslavsky *et al* reported PD-L1 expression on platelets in metastatic cancer patients and demonstrated that platelets bind to cancer cells supporting the theory that platelets provide an immune escape mechanism [35]. In this paper, we provide evidence of substantial inter- and intra-patient heterogeneity of platelet-PD-L1 expression, consistent with the clinical observation of variable sensitivity of patient responses to immune checkpoint inhibition therapy to date [3, 36].

Taken together, these results strongly suggest that CTC PD-L1 and platelet PD-L1 expression are not due to technical or clinical artifacts, and might have clinical relevance in the treatment of patients with cancer. Several different putative predictive factors for anti-PD-L1 and anti-PD-1 therapies have been reported, including expression of PD-L1 itself, either on cancer cells, tissue or circulating immune effector cells [37, 38]. Presence of CTC in NCSLC patients receiving ICPi have been reported as a predictor of worse response [39]. Furthermore, CTC PD-L1 has been reported to be an independent predictor of improved progression free survival in MBC patients who did not receive ICPi [26] as well as in melanoma patients starting pembrolizumab [19]. In addition to platelet PD-L1 identified in patients with metastatic cancer [35], platelet PD-L1 expression was also recently reported in four patients with lung cancer who benefited from atezolizumab [29]. Of interest, the association of CTC PD-L1 with CDK4/6 treatment in our study coincides with a report that CDK4/6 inhibitors upregulate PD-L1 expression [40, 41]. Therefore, determination and monitoring of CTC PD-L1 in patients receiving these agents provides insight into combination ICPi and CDK4/6 inhibitors. Additionally, studies have shown that radiotherapy transiently increases PD-L1 expression on CTC in NSCLC, suggesting ICPi can be used in conjunction with radiotherapy for added efficacy as well as further highlighting the importance of monitoring CTC PD-L1 [42].

Of note, the remarkable inter-patient heterogeneity suggests a potential biological, and perhaps pharmacodynamic, role for both CTC and platelet PD-L1 expression. The ease of identifying and quantitating CTC levels, CTC PD-L1 and platelet PD-L1 expression on a single analytical platform (CellSearch^®^) should facilitate further research in this area. In this regard, we are planning a pilot clinical study to determine if CTC or platelet PD-L1 expression might have a role in directing treatment with ICPi therapies.

In summary, we have demonstrated that PD-L1 expression can be reliably and quantitatively evaluated on CTC and platelets using the FDA-cleared CellSearch^®^ assay in MBC patients. We speculate that CTC and/or platelet PD-L1 expression could predict benefit from ICPi therapies, particularly those directed towards the PD-1/PD-L1 pathway. Prospective studies of patients receiving anti-PD-1/PD-L1 therapies are warranted to further explore PD-L1 on both CTC and platelets in order to correlate findings with response rates and progression free survival to validate the use of PD-L1 as a predictive biomarker for liquid biopsies.

## Supporting information

S1 File Supplementary material. (PDF)

S1 Fig REMARK diagram of blood samples from MBC patients used to evaluate CTC PD-L1 and platelet PD-L1 expression. (PDF)

S2 Fig Semi-quantitative scale of PD-L1 expression on cultured human breast cancer cells spiked in healthy blood and retrieved using the CellSearch® system. Cultured human breast cancer cells with known expression of PD-L1 were spiked into normal human whole blood and processed using CellSearch®. The respective columns represent fluorescent-generated images from each of the filters (CK, DAPI, CD45), plus a composite image, arranged as thumbnail images in a gallery format. The last column represents fluorescent imaging with a labeled antibody against PD-L1. These were visually scored as 0, 1+, or 2+. All cells scored as 1+ and 2+ were arbitrarily considered positive for PD-L1 staining. See S1 File for details.
(PDF)

S3 Fig Platelet PD-L1 co-staining. A. CellSearch® thumbnail images from a patient with PD-L1 positive CTC as well as PD-L1 positive platelets. Orange boxes in composite column = intact CTC according to classic CellSearch® algorithm. All boxes in the PD-L1 column display apparent platelet staining, whether CTC are present or not. B. Contents from the CellSearch cartridge from a patient with CellSearch PD-L1 positive platelets, illustrated in panel A, were extracted and stained with platelet specific antibodies. A colored composite image of DAPI, PD-L1, and platelet specific markers CD-42b/CD-41(APC/Cy7 conjugated) along with corresponding monochrome images for PD-L1, platelets, DAPI, and cytokeratin are shown. On the colored composite image, yellow arrows point to PD-L1 positive platelets co-stained with additional antibodies for platelet specific markers CD-42b/CD-41; dashed white arrows point to platelets stained positive for platelet specific markers CD-42b/CD-41 and negative for PD-L1; red arrow points to nucleated (DAPI positive) cells, either circulating tumor cells or white blood cells, that are also positive for PD-L1. C. CellSearch® thumbnail images from a patient with PD-L1 negative CTC as well as <100 PD-L1 positive platelets. D. Contents from the CellSearch cartridge from a patient with CellSearch PD-L1 negative platelets, illustrated in panel C, were extracted and stained with platelet specific antibodies. A colored composite image of DAPI, PD-L1, and platelet specific markers CD-42b/CD-41(APC/Cy7 conjugated) along with corresponding monochrome images for PD-L1, platelets, DAPI, CD-45 and cytokeratin are shown. On the colored composite image, yellow arrows point to PD-L1 positive platelets co-stained with additional antibodies for platelet specific markers CD-42b/CD-41; dashed white arrows point to platelets stained positive for platelet specific markers CD-42b/CD-41 and negative for PD-L1; red arrow points to nucleated (DAPI positive) cells, either circulating tumor cells or white blood cells, that are also positive for PD-L1.
(PDF)

S4 Fig Effect of fixative in whole blood collection tubes on platelet PD-L1 staining. Whole blood drawn from 13 patients with MBC was collected into 10 cc vacutainers containing formalin-based fixative (CellSave tubes) or containing EDTA but no fixative and processed in the CellSearch®, as described in Methods. The 5th column represents fluorescent staining for anti-PDL1. A. Image illustrating CellSearch platelet PD-L1 positivity in patient sample for which whole blood was collected into CellSave tube containing fixative. B. Image illustrating CellSearch platelet PD-L1 positivity in the same patient sample for which whole was collected into EDTA tube not containing fixative.
(PDF)

S5 Fig Distribution of PD-L1 positive platelets across CellSearch cartridge. The CellSearch cartridge is divided into 175 frames, each frame can be observed by a single fluorescent marker. A-C Three frames selected to view the PE-labeled PD-L1 fluorescence (#69, #94, #108 respectively) at different locations within a single CellSearch cartridge for a patient with PD-L1 positive platelets. The grid located at the top of each image indicates the entire CellSearch cartridge divided into 175 frames. The orange box in the grid indicates the location of the frame in view, highlighted with red arrow. Yellow boxes within frame #94 and #108 indicate cells detected by the CellSearch algorithm, which are represented as enlarged, single cell images in the thumbnail galleries.
(PDF)

S6 Fig PD-L1 sensitivity and specificity of antibody clone 29E.2A3 for PD-L1. A.Western blot of cell lysates from cultured human breast cancer cell lines known to express (MDA-MB-231) or not to express PD-L1 (MCF-7) using E1L3N Rabbit mAb, confirming the PD-L1 status of these two cell lines. B. PD-L1 protein expression by Western blot of lysates of MDA-MB-231 cell line (PD-L1 positive) treated with siRNA against PD-L1. PD-L1 expression was determine with E1L3N Rabbit mAb. Clones of MDA-MB-231 cells treated with siRNA against PD-L1 (s26547, s26548, and s26549) or with scrambled or nonsense probes, confirming the success of the PD-L1 knockdown process. C. PD-L1 gene expression analysis of MDA-MB-231 cell lines treated with siRNA against PD-L1. Clones of MDA-MB-231 cells treated with siRNA against PD-L1 as described in B above, confirming the success of the PD-L1 knock down process. (■) Primer Pair 3 PD-L1; (■) Primer Pair 2 PD-L1; (■) Primer Pair 1 PD-L1. D. Thumbnail gallery images of PD-L1 protein expression on MDA-MB-231 cell lines (wild type and PD-L1 siRNA treated cell lines when processed by CellSearch® (see details in B above). This illustration is from an MDA-MB-231 clone treated with siRNA against PD-L1 (siRNA s26547). The 5th column represents fluorescent staining for PD-L1. 0, 1+ and 2+ represent visual scoring for PD-L1 as described. E. PD-L1 protein expression when processed by CellSearch® on MDA-MB-231 cell lines (wild type and PD-L1 siRNA treated cell lines (see details in B above). Graphic representation of fluorescent staining for PD-L1 when visually scored as 0-2+ (see S1 File for details). Wild type MDA-MB-231 and clones treated with scrambled and nonsense probes have high percentage (≥90%) of 2+ fluorescent staining for PD-L1. Clones treated with siRNA (s26547, s26548, and s26549) have <25% 2+, and ≥75% staining with 0-1+. These data are concordant with B and C, and strongly suggest that clone 29E.2A3 is staining PD-L1 protein.
(PDF)

S7 Fig PD-L1 expression on cultured breast cancer cell lines spiked into healthy donor whole blood and processed through CellSearch®. A. MDA-MB-231: 97.5% of cells were strongly PD-L1 positive (2+), 2.5% of cells were PD-L1 negative (0). B. MDA-MB-468: 7.4% of cells were strongly PD-L1 positive (2+), 6.6% of cells were weakly PD-L1 positive (1+), and 86% of cells were PD-L1 negative (0). C. Sk-Br-3: 3% of cells were strongly PD-L1 positive (2+), 6% of cells were weakly PD-L1 positive (1+), and 91% of cells were PD-L1 negative (0). D. BT-474: 0.01% of cells were weakly PD-L1 positive (1+), and 99.99% of cells were PD-L1 negative (0). E. MCF-7: 100% of cells were PD-L1 negative (0).
(PDF)

S8 Fig Semi-quantitative scale of platelets PD-L1 positivity per CellSearch® frame. A. CellSearch® thumbnail images, with each row representing a single cell and each column representing fluorescence of protein markers. The 5th column illustrates fluorescent staining for PD-L1. B. A single frame within a CellSearch® cartridge. Magnification of a single frame (highlighted with red), one of the 175 frames. C. Examples of CellSearch® frames with platelet PD-L1 staining count of >1,000, 100-1,000, <100, and 0. Samples with an average number of platelet PD-L1/3 frames of >1,000 and 100-1,000 PD-L1 were arbitrarily designated positive for PD-L1 staining. CellSearch® frames with 0 and <100 PD-L1 positive platelets per frame were designated as negative for PD-L1 staining.
(PDF)

S1 Table PD-L1 expression as determined by CellSearch® in cultured cell lines. The characteristics for ER, PgR, HER2 expression as well as cell culture media information are provided.
(PDF)

S2 Table Platelet PD-L1 expression in Fixed versus non-fixed whole blood. (PDF)

S3 Table Platelet PD-L1 expression according to platelet count in CellSearch® cartridges. (PDF)

S4 Table Patient demographic characteristics. (PDF)

S5 Table Univariable association of factors of interest with CTC PD-L1 positive rate. (PDF)

S6 Table Multivariable results of factors of interest with CTC PD-L1 positivity rate. (PDF)

S7 Table Univariable association of factors of interest with Platelet PD-L1 positivity. (PDF)

S8 Table Multivariable results of factors of interest with Platelet PD-L1 positivity. (PDF)

S9 Table Serial timepoints CTC, Platelet PD-L1, treatment, days between serial timepoints. (PDF)
