# Supplementary material for: PD-L1 expression on circulating tumor cells and platelets in patients with metastatic breast cancer
Source: PLoS One. 2021 Nov 15;16(11):e0260124. doi: 10.1371/journal.pone.0260124 (PMC8592410; doi:10.1371/journal.pone.0260124)
Supplement: S1 File — (PDF) [file pone.0260124.s001.pdf]

SUPPLEMENTARY MATERIAL

Patient selection (S1 Fig, S4 Table)

Eligibility and entry criteria for enrollment into the study are described in Methods. **S1 Fig** describes distribution of the patients and **S4 Table** describes the patient demographics.

Definition of CTC PD-L1 staining scale (S2 Fig)

After processing circulating tumor cells (CTC) through CellSearch®, and staining for programed death ligand 1 (PD-L1) (see Methods), CTC were visually determined to have relative PD-L1 staining according to a scale similar to what we have previously published for other markers, ranging from 0-3+ [1, 2]. It should be noted that we have not observed any cultured breast cancer cells or patient circulating tumor cells that stained 3+ for PD-L1. Therefore, we considered CTC PD-L1 staining of 1+ or 2+ as positive. This scale depends on both the relative intensity of the CTC staining and the relative clarity of the background, ranging from dark black to gray/white. The latter occurs when the laser scanner used to identify events amplifies the signal to determine whether the event is or is not fluorescing. Thus, an event (CTC) that is very bright will have a very dark background, and is designated 3+, whereas an event that is dim with a gray/white background is 0. **S2 Fig** provides images from specimens that contain variable CTC PD-L1 staining as an illustration CTC PD-L1 that stained 0, 1+, and 2+.

Co-staining of platelets and PD-L1 (S3 Fig)

In order to determine that the objects staining for PD-L1 were platelets, CellSearch® enriched contents from whole blood (WB), which had been stained with DAPI and anti-CD45, CK and PD-L1 was removed from the CellSearch® cartridge and stained for platelet-specific markers, CD-41 and CD-42b, and spun onto poly-lysine coated slides. Specimens from patients in which we observed apparent platelet

PD-L1 positivity in the CellSearch® system did, indeed, have co-staining of anti-platelet antibodies and anti PD-L1 on the glass slides (**S3A-B Figs**). Additionally, a patient with <100 PD-L1 positive platelets was also co-stained with CD-41 and CD-42b antibodies after processing through CellSearch. PD-L1 positive and PD-L1 negative platelets are present (**S3C-D Figs**).

Fixed vs. nonfixed blood (S4 Fig, S2 Table)

To determine if the preservative fixative in CellSave tubes impacted the presence of PD-L1 positive platelets in patient samples, we drew WB in both CellSave and ethylenediaminetetraacetic acid (EDTA) non-fixative containing tubes from 13 patients, processed the blood in parallel using the CellSearch® assay, and stained for PD-L1 (**S4 Fig**). The presence or absence of PD-L1 positive platelets was concordant (+/+ = 9; -/- = 3; +/- = 0; -/+ = 1) in 12/13 patient samples (**S2 Table**). One patient stained positive for platelet PD-L1 expression in WB drawn into EDTA tubes, but did not have platelet PD-L1 positive in WB drawn into CellSave tubes (**S2 Table**).

Platelet PD-L1 expression according to platelet count in CellSearch® cartridges (S3 Table)

Routine clinical complete blood count (CBC) platelet levels determined on the same day as research blood collection ranged from 159,000 to 425,000/ul (**S3 Table**). Platelet counts carried over into the CellSearch® cartridge for these patients ranged from 1,000-3,000 platelets/ul of enriched CellSearch product, and were not related to the CBC-platelet count (**S3 Table**). Four of these seven patients had <100, two had 100-1,000 and one had >1,000 PD-L1 positive platelet staining, determined from CellSearch® PD-L1 analysis, which, again, were independent of CBC-platelet or CellSearch®-cartridge carry-over platelet levels. These results confirmed that the number of PD-L1 positive platelets per CellSearch® frame were independent of the number of platelets assessed in CBC as well as the number of platelets carried over during the CellSearch® enrichment process.

PD-L1 antibody specificity (S6 and S7 Figs, S1 Table)

The Biolegend PE-conjugated PD-L1 antibody, clone 29E.293, which we have used in the CellSearch® system for CTC PD-L1 expression analysis, has an affinity for PD-L1 only in the native conformation. Due to this limitation, we ensured the specificity of this antibody for PD-L1 indirectly by siRNA knockdown of PD-L1 in MDA-MB-231 cells. Initially, both MDA-MB-231 and MCF-7 cell lines were tested for PD-L1 expression by Western blot using a separate antibody from 29E.293, designated as clone E1L3N (Cell Signaling Technologies, Danvers, MA). Clone E1L3N retains binding to PD-L1 after denaturation. As expected, MDA-MB-231 cells had high expression of PD-L1 and were subsequently selected for siRNA knockdown experiments (**S6A Fig**).

Each of the three siRNA's selected bind to a different region along the PD-L1 transcript. Five separate conditions of MDA-MB-231 cells were cultured in parallel: untreated, mock transfected, siRNA s26547, siRNA s26548, and siRNA s26549. The untreated and the nonsense control cell lines maintained high levels of PD-L1 expression by Western blot (**S6B Fig**). However, all three PD-L1 knockdown cell lines were negative for PD-L1 expression by Western blot analysis (**S6B Fig**).

To further analyze the efficacy of the knockdown of PD-L1 in MDA-MB-231 cells, we examined PD-L1 message expression. Cells transfected with the nonsense control did not exhibit a reduction in PD-L1 expression (**S6C Fig**). Compared to untreated cells, s26547 had a 75% reduction in PD-L1 (**S6C Fig**). Similarly, s26548 had an 80% reduction in PD-L1 expression compared to untreated cells. The s26549 transfected cells had a 40% reduction in PD-L1 expression (**S6C Fig**). These results confirmed siRNA knockdown of PD-L1 expression in transfected cell lines.

Having proven that the respective cell lines in our laboratory were positive (MDA-MB-231) or negative (MCF-7, SK-Br-3, BT-474), and that MDA-MB-231 siRNA knockdown cells no longer expressed PD-L1, we investigated whether the PD-L1 antibody clone 29E.293 behaved as expected in the CellSearch® system. Wild type and transfected cells were harvested and spiked into 7.5ml of healthy

donor blood. Spiked WB was processed using the CellSearch® platform staining for CK, DAPI, CD-45, and PD-L1. After processing through CellSearch®, "spiked CTC" were enumerated and CTC PD-L1 expression was assessed (**S6D Fig**). Expression of PD-L1 on tumor cells was classified as 0 (no PD-L1 expression), 1+ (low PD-L1 expression), and 2+ (representing high PD-L1 expression), in a manner similar to the CTC-protein expression scale we have previously reported for ER, BCL2, HER2, Ki67, and M30, as illustrated in **S2 Fig** [1, 2].

We then interrogated MDA-MB-231 PD-L1 knockdown cells, as well as those treated with the nonsense control, for staining with PD-L1 antibody clone 29E.293. In this case, 46% and 36.5% of the s26547 knockdown cells were completely negative or 1+ (weakly positive), respectively. Only 17.5% of s26547 knockdown cells were 2+ (strongly positive). In contrast, 90.5% of the nonsense-transfected control cells stained 2+ (strongly positive), whereas only 8% were 1+ (weakly positive) (**S6E Fig**). Similar results were found for both s26548 and s26549 (**S6E Fig**). Taken together, these data confirmed that monoclonal antibody clone 29E.293 is specific for PD-L1 expression, and therefore appropriate for use in the CellSearch® system.

To further determine the sensitivity and specificity of PD-L1 staining with antibody clone 29E.293, we then stained a series of different cultured human breast cancer cell lines with known PD-L1 expression [3]. As expected, wild-type MD-MB-231 had the highest PD-L1 expression with 97.5% of cells 2+ (**S7A Fig**), whereas the remaining 2.5% of cells were completely negative (0) for PD-L1 expression. Of the wild-type MDA-MB-468 cells (**S7B Fig**), 7.4% stained as 2+, 6.6% stained as 1+, and 86% of cells were 0. Sk-Br-3 cells, 3% stained as 2+, 6% stained as 1+, and 91% of cells were 0 for PD-L1 expression (**S7C Fig**), and both BT-474 and MCF-7 cells were completely negative (**S7D-E Figs**).

Quantifying PD-L1 positive platelets in CellSearch® (S8 Fig)

Each CellSearch® cartridge was scanned using the CellTracks analyzer. Single cells are presented individually in thumbnail galleries (**S8A Fig**). Alternatively, images from the cartridge can be viewed by

frames. Each CellSearch® cartridge is divided into 175 frames (**S8B Fig**). Each frame can be assessed for each fluorochrome (DAPI, FITC, APC, or PE) individually. Platelets were evenly distributed between frames; therefore, three frames were randomly selected from the 175 total frames to assess for the number of platelet PD-L1 positivity (**S8B Fig**). An average platelet count per three CellSearch frames was calculated for each specimen. We then generated a semi-quantitative scale of 0, <100, 100-1000, and >1,000 PD-L1 positive platelet count per three CellSearch frames of the CellSearch® cartridge. We arbitrarily considered platelet PD-L1 0-99/frame as negative and ≥100/frame as positive (**S8C Fig**).

Association of CTC PD-L1 and platelet PD-L1 expression with clinical and pathological factors (S4-8 Tables)

Each patient's medical chart was reviewed with a prospective list of possible factors that might affect PD-L1 expression. **S4 Table** provides the relative status of these factors. **S5 and S6 Tables** provide uni- and multi-variable associations, respectively, of each factor with CTC PD-L1 and **S7 and S8 Tables** provide uni- and multi-variable associations, respectively, with platelet PD-L1 expression. CTC PD-L1 expression was determined visually as described above and considered positive, if it was 1+ or 2+. Platelet PD-L1 expression was determined as described above and ≥100/frame was considered positive. Associations were considered significant if the p-value describing the relative Rate Ratio for CTC PD-L1 and Odds Ratio for Platelet PD-L1 was <0.001 for univariable analyses and <0.05 for multivariable analyses.

REFERENCES

1. Paoletti C, Muniz MC, Thomas DG, Griffith KA, Kidwell KM, Tokudome N, et al. Development of circulating tumor cell-endocrine therapy index in patients with hormone receptor-positive breast cancer. Clin Cancer Res. 2015;21(11):2487-98. doi: 10.1158/1078-0432.CCR-14-1913. PubMed PMID: 25381338; PubMed Central PMCID: PMCPMC5516625.

2. Paoletti C, Larios JM, Muniz MC, Aung K, Cannell EM, Darga EP, et al. Heterogeneous estrogen receptor expression in circulating tumor cells suggests diverse mechanisms of fulvestrant resistance. Mol Oncol. 2016;10(7):1078-85. Epub 2016/05/15. doi: 10.1016/j.molonc.2016.04.006. PubMed PMID: 27178224; PubMed Central PMCID: PMCPMC5423180.

3. Mittendorf EA, Philips AV, Meric-Bernstam F, Qiao N, Wu Y, Harrington S, et al. PD-L1 expression in triple-negative breast cancer. Cancer Immunol Res. 2014;2(4):361-70. Epub 2014/04/26. doi: 10.1158/2326-6066.CIR-13-0127. PubMed PMID: 24764583; PubMed Central PMCID: PMCPMC4000553.
