# Supplementary material for: PD-L1 expression on circulating tumor cells and platelets in patients with metastatic breast cancer
Source: PLoS One. 2021 Nov 15;16(11):e0260124. doi: 10.1371/journal.pone.0260124 (PMC8592410; doi:10.1371/journal.pone.0260124)
Supplement: S1 Fig — (PDF) [file pone.0260124.s002.pdf]

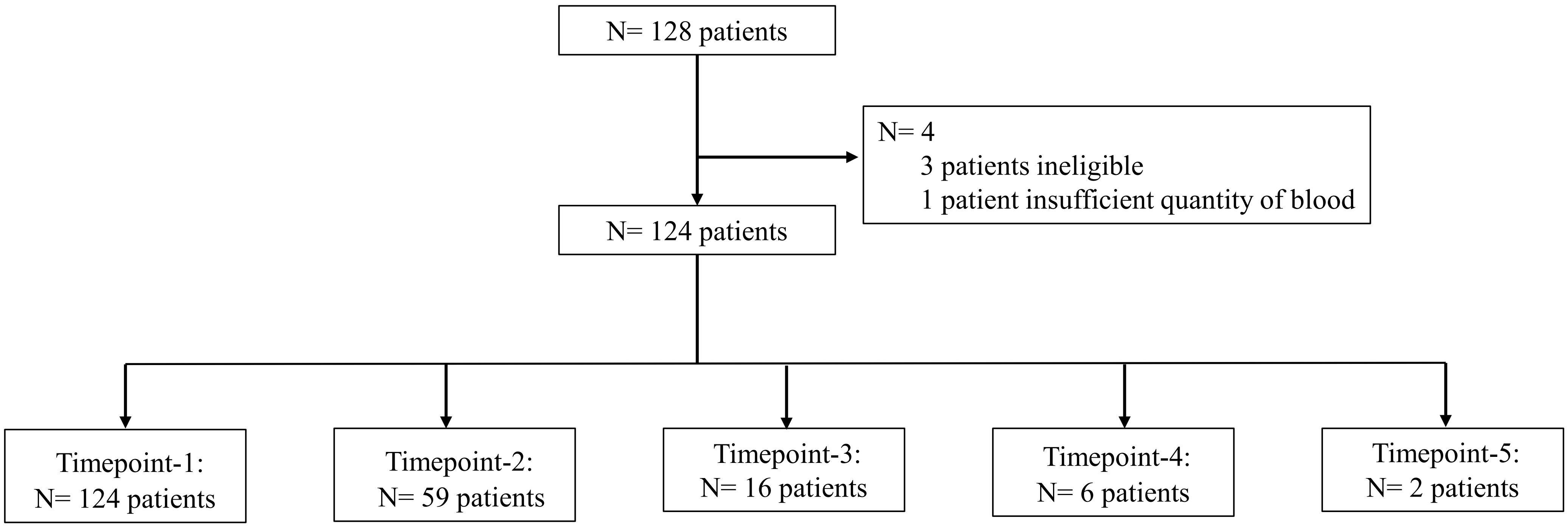

S1 Fig. REMARK diagram of blood samples from MBC patients used to evaluate CTC PD-L1 and platelet PD-L1 expression.
