# Supplementary material for: PD-L1 expression on circulating tumor cells and platelets in patients with metastatic breast cancer
Source: PLoS One. 2021 Nov 15;16(11):e0260124. doi: 10.1371/journal.pone.0260124 (PMC8592410; doi:10.1371/journal.pone.0260124)
Supplement: S2 Fig — Cultured human breast cancer cells with known expression of PD-L1 were spiked into normal human whole blood and processed using CellSearch®. The respective columns represent fluorescent-generated images from each of the filters (CK, DAPI, CD45), plus a composite image, arranged as thumbnail images in a gallery format. The last column represents fluorescent imaging with a labeled antibody against PD-L1. These were visually scored as 0, 1+, or 2+. All cells scored as 1+ and 2+ were arbitrarily considered positive for PD-L1 staining. See S1 File for details. (PDF) [file pone.0260124.s003.pdf]

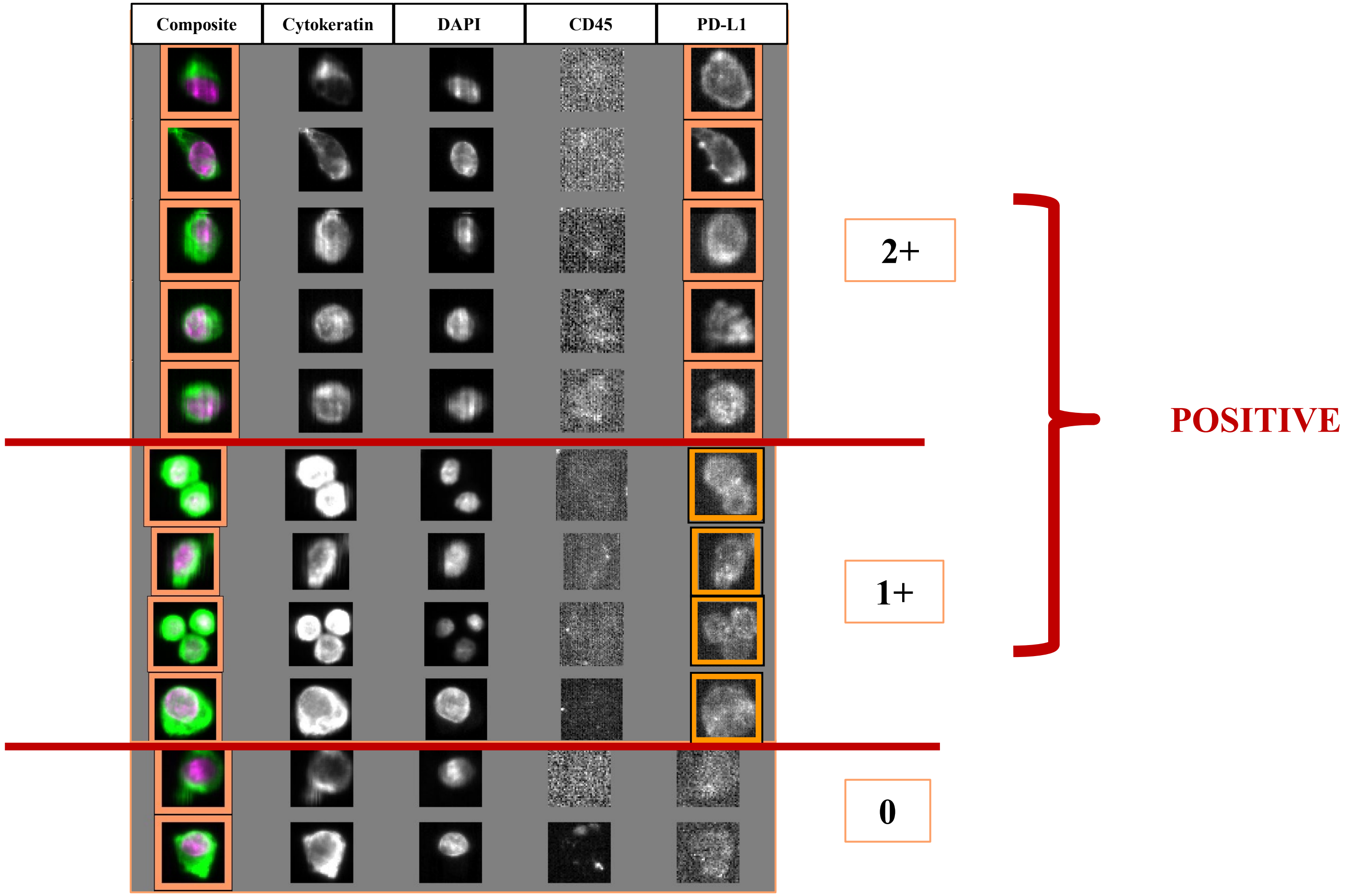

S2 Fig. Semi-quantitative scale of PD-L1 expression on cultured human breast cancer cells spiked in healthy blood and retrieved using the CellSearch® system. Cultured human breast cancer cells with known expression of PD-L1 were spiked into normal human whole blood and processed using CellSearch®. The respective columns represent fluorescent-generated images from each of the filters (CK, DAPI, CD45), plus a composite image, arranged as thumbnail images in a gallery format. The last column represents fluorescent imaging with a labeled antibody against PD-L1. These were visually scored as 0, 1+, or 2+. All cells scored as 1+ and 2+ were arbitrarily considered positive for PD-L1 staining. See S1 File for details.
