# Supplementary material for: PD-L1 expression on circulating tumor cells and platelets in patients with metastatic breast cancer
Source: PLoS One. 2021 Nov 15;16(11):e0260124. doi: 10.1371/journal.pone.0260124 (PMC8592410; doi:10.1371/journal.pone.0260124)
Supplement: S3 Fig — A. CellSearch® thumbnail images from a patient with PD-L1 positive CTC as well as PD-L1 positive platelets. Orange boxes in composite column = intact CTC according to classic CellSearch® algorithm. All boxes in the PD-L1 column display apparent platelet staining, whether CTC are present or not. B. Contents from the CellSearch cartridge from a patient with CellSearch PD-L1 positive platelets, illustrated in panel A, were extracted and stained with platelet specific antibodies. A colored composite image of DAPI, PD-L1, and platelet specific markers CD-42b/CD-41(APC/Cy7 conjugated) along with corresponding monochrome images for PD-L1, platelets, DAPI, and cytokeratin are shown. On the colored composite image, yellow arrows point to PD-L1 positive platelets co-stained with additional antibodies for platelet specific markers CD-42b/CD-41; dashed white arrows point to platelets stained positive for platelet specific markers CD-42b/CD-41 and negative for PD-L1; red arrow points to nucleated (DAPI positive) cells, either circulating tumor cells or white blood cells, that are also positive for PD-L1. C. CellSearch® thumbnail images from a patient with PD-L1 negative CTC as well as <100 PD-L1 positive platelets. D. Contents from the CellSearch cartridge from a patient with CellSearch PD-L1 negative platelets, illustrated in panel C, were extracted and stained with platelet specific antibodies. A colored composite image of DAPI, PD-L1, and platelet specific markers CD-42b/CD-41(APC/Cy7 conjugated) along with corresponding monochrome images for PD-L1, platelets, DAPI, CD-45 and cytokeratin are shown. On the colored composite image, yellow arrows point to PD-L1 positive platelets co-stained with additional antibodies for platelet specific markers CD-42b/CD-41; dashed white arrows point to platelets stained positive for platelet specific markers CD-42b/CD-41 and negative for PD-L1; red arrow points to nucleated (DAPI positive) cells, either circulating tumor cells or white blood [file pone.0260124.s004.pdf]

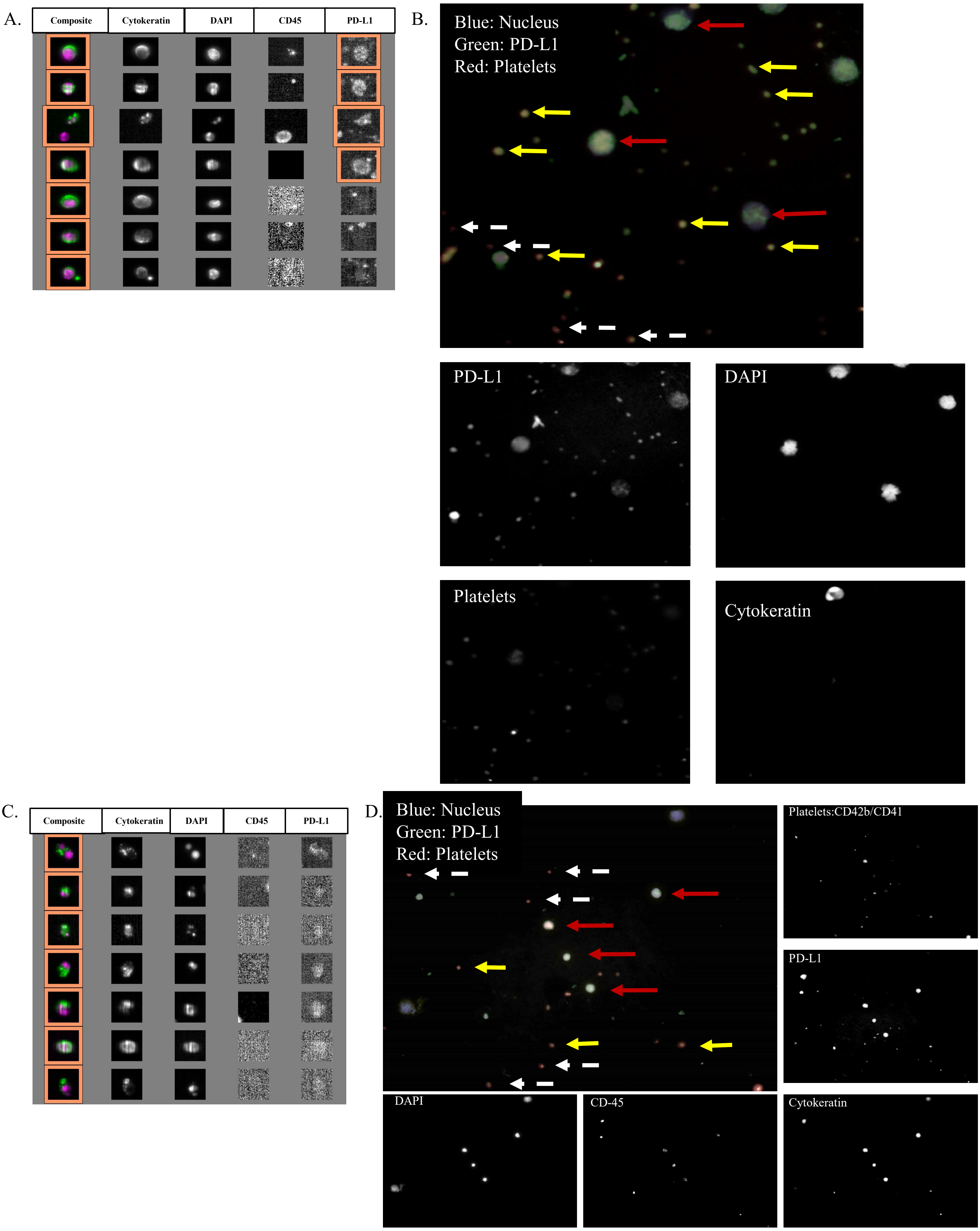

S3 Fig. Platelet PD-L1 Co-staining. A. CellSearch® thumbnail images from a patient with PD-L1 positive CTC as well as PD-L1 positive platelets. Orange boxes in composite column = intact CTC according to classic CellSearch® algorithm. All boxes in the PD-L1 column display apparent platelet staining, whether CTC are present or not. B. Contents from the CellSearch cartridge from a patient with CellSearch PD-L1 positive platelets, illustrated in panel A, were extracted and stained with platelet specific antibodies. A colored composite image of DAPI, PD-L1, and platelet specific markers CD-42b/CD-41(APC/Cy7 conjugated) along with corresponding monochrome images for PD-L1, platelets, DAPI, and cytokeratin are shown. On the colored composite image, yellow arrows point to PD-L1 positive platelets co-stained with additional antibodies for platelet specific markers CD-42b/CD-41; dashed white arrows point to platelets stained positive for platelet specific markers CD-42b/CD-41 and negative for PD-L1; red arrow points to nucleated (DAPI positive) cells, either circulating tumor cells or white blood cells, that are also positive for PD-L1. C. CellSearch® thumbnail images from a patient with PD-L1 negative CTC as well as <100 PD-L1 positive platelets. D. Contents from the CellSearch cartridge from a patient with CellSearch PD-L1 negative platelets, illustrated in panel C, were extracted and stained with platelet specific antibodies. A colored composite image of DAPI, PD-L1, and platelet specific markers CD-42b/CD-41(APC/Cy7 conjugated) along with corresponding monochrome images for PD-L1, platelets, DAPI, CD-45 and cytokeratin are shown. On the colored composite image, yellow arrows point to PD-L1 positive platelets co-stained with additional antibodies for platelet specific markers CD-42b/CD-41; dashed white arrows point to platelets stained positive for platelet specific markers CD-42b/CD-41 and negative for PD-L1; red arrow points to nucleated (DAPI positive) cells, either circulating tumor cells or white blood cells, that are also positive for PD-L1.
