# Supplementary material for: PD-L1 expression on circulating tumor cells and platelets in patients with metastatic breast cancer
Source: PLoS One. 2021 Nov 15;16(11):e0260124. doi: 10.1371/journal.pone.0260124 (PMC8592410; doi:10.1371/journal.pone.0260124)
Supplement: S4 Fig — Whole blood drawn from 13 patients with MBC was collected into 10 cc vacutainers containing formalin-based fixative (CellSave tubes) or containing EDTA but no fixative and processed in the CellSearch®, as described in Methods. The 5th column represents fluorescent staining for anti-PDL1. A. Image illustrating CellSearch platelet PD-L1 positivity in patient sample for which whole blood was collected into CellSave tube containing fixative. B. Image illustrating CellSearch platelet PD-L1 positivity in the same patient sample for which whole was collected into EDTA tube not containing fixative. (PDF) [file pone.0260124.s005.pdf]

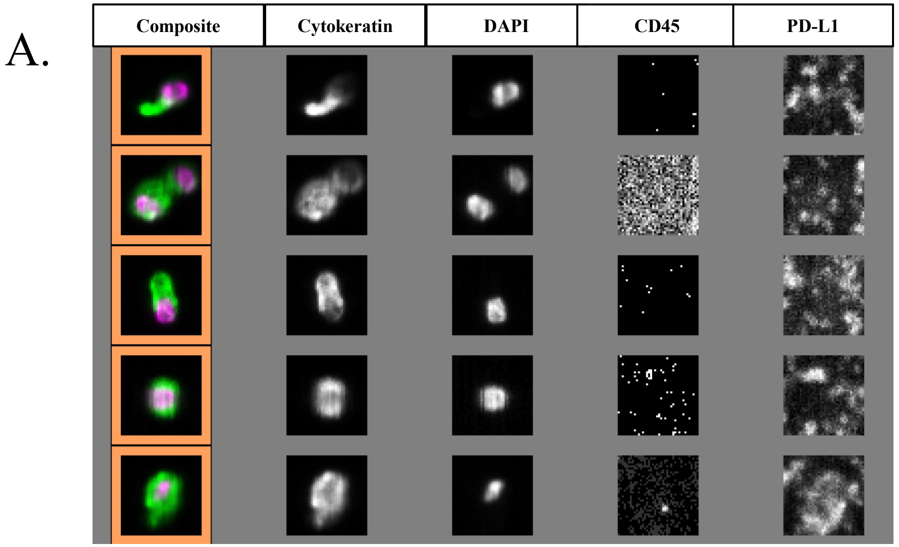

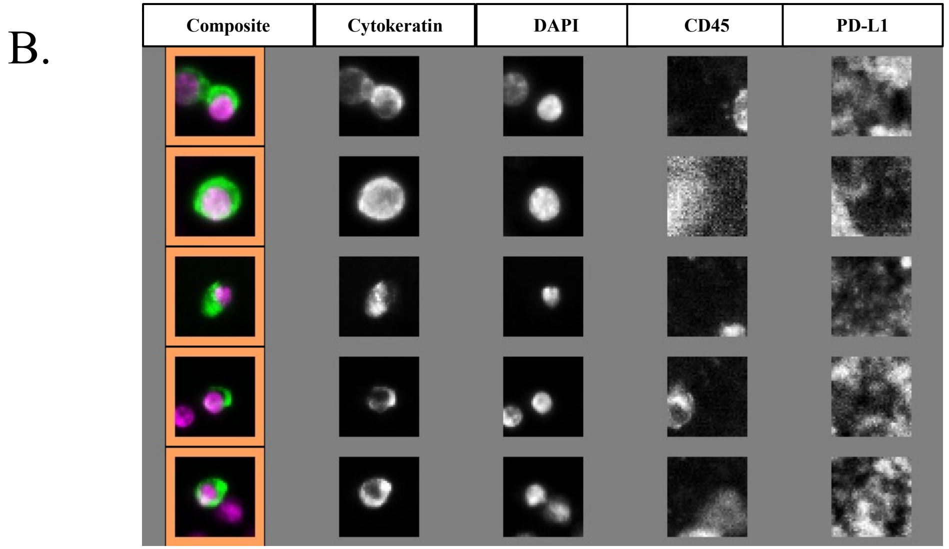

S4 Fig. Effect of fixative in whole blood collection tubes on platelet PD-L1 staining. Whole blood drawn from 13 patients with MBC was collected into 10 cc vacutainers containing formalin-based fixative (CellSave tubes) or containing EDTA but no fixative and processed in the CellSearch®, as described in Methods. The 5th column represents fluorescent staining for anti-PDL1. A. Image illustrating CellSearch platelet PD-L1 positivity in patient sample for which whole blood was collected into CellSave tube containing fixative. B. Image illustrating CellSearch platelet PD-L1 positivity in the same patient sample for which whole was collected into EDTA tube not containing fixative.
