# Supplementary material for: PD-L1 expression on circulating tumor cells and platelets in patients with metastatic breast cancer
Source: PLoS One. 2021 Nov 15;16(11):e0260124. doi: 10.1371/journal.pone.0260124 (PMC8592410; doi:10.1371/journal.pone.0260124)
Supplement: S5 Fig — The CellSearch cartridge is divided into 175 frames, each frame can be observed by a single fluorescent marker. A-C Three frames selected to view the PE-labeled PD-L1 fluorescence (#69, #94, #108 respectively) at different locations within a single CellSearch cartridge for a patient with PD-L1 positive platelets. The grid located at the top of each image indicates the entire CellSearch cartridge divided into 175 frames. The orange box in the grid indicates the location of the frame in view, highlighted with red arrow. Yellow boxes within frame #94 and #108 indicate cells detected by the CellSearch algorithm, which are represented as enlarged, single cell images in the thumbnail galleries. (PDF) [file pone.0260124.s006.pdf]

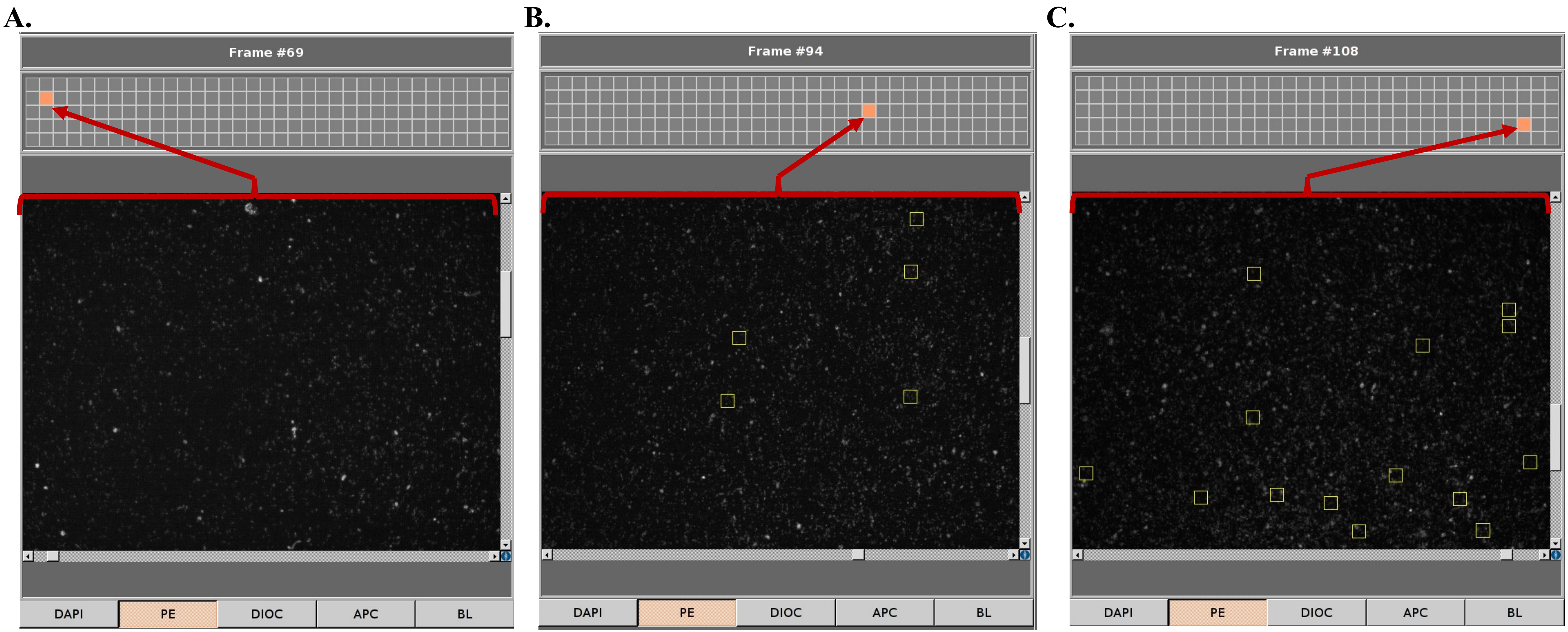

S5 Fig. Distribution of PD-L1 positive platelets across CellSearch cartridge. The CellSearch cartridge is divided into 175 frames, each frame can be observed by a single fluorescent marker. A-C Three frames selected to view the PE-labeled PD-L1 fluorescence (#69, #94, #108 respectively) at different locations within a single CellSearch cartridge for a patient with PD-L1 positive platelets. The grid located at the top of each image indicates the entire CellSearch cartridge divided into 175 frames. The orange box in the grid indicates the location of the frame in view, highlighted with red arrow. Yellow boxes within frame #94 and #108 indicate cells detected by the CellSearch algorithm, which are represented as enlarged, single cell images in the thumbnail galleries.
