# Supplementary material for: PD-L1 expression on circulating tumor cells and platelets in patients with metastatic breast cancer
Source: PLoS One. 2021 Nov 15;16(11):e0260124. doi: 10.1371/journal.pone.0260124 (PMC8592410; doi:10.1371/journal.pone.0260124)
Supplement: S6 Fig — A.Western blot of cell lysates from cultured human breast cancer cell lines known to express (MDA-MB-231) or not to express PD-L1 (MCF-7) using E1L3N Rabbit mAb, confirming the PD-L1 status of these two cell lines. B. PD-L1 protein expression by Western blot of lysates of MDA-MB-231 cell line (PD-L1 positive) treated with siRNA against PD-L1. PD-L1 expression was determine with E1L3N Rabbit mAb. Clones of MDA-MB-231 cells treated with siRNA against PD-L1 (s26547, s26548, and s26549) or with scrambled or nonsense probes, confirming the success of the PD-L1 knockdown process. C. PD-L1 gene expression analysis of MDA-MB-231 cell lines treated with siRNA against PD-L1. Clones of MDA-MB-231 cells treated with siRNA against PD-L1 as described in B above, confirming the success of the PD-L1 knock down process. (■) Primer Pair 3 PD-L1; (■) Primer Pair 2 PD-L1; (■) Primer Pair 1 PD-L1. D. Thumbnail gallery images of PD-L1 protein expression on MDA-MB-231 cell lines (wild type and PD-L1 siRNA treated cell lines when processed by CellSearch® (see details in B above). This illustration is from an MDA-MB-231 clone treated with siRNA against PD-L1 (siRNA s26547). The 5th column represents fluorescent staining for PD-L1. 0, 1+ and 2+ represent visual scoring for PD-L1 as described. E. PD-L1 protein expression when processed by CellSearch® on MDA-MB-231 cell lines (wild type and PD-L1 siRNA treated cell lines (see details in B above). Graphic representation of fluorescent staining for PD-L1 when visually scored as 0-2+ (see S1 File for details). Wild type MDA-MB-231 and clones treated with scrambled and nonsense probes have high percentage (≥90%) of 2+ fluorescent staining for PD-L1. Clones treated with siRNA (s26547, s26548, and s26549) have <25% 2+, and ≥75% staining with 0-1+. These data are concordant with B and C, and strongly suggest that clone 29E.2A3 is staining PD-L1 protein. (PDF) [file pone.0260124.s007.pdf]

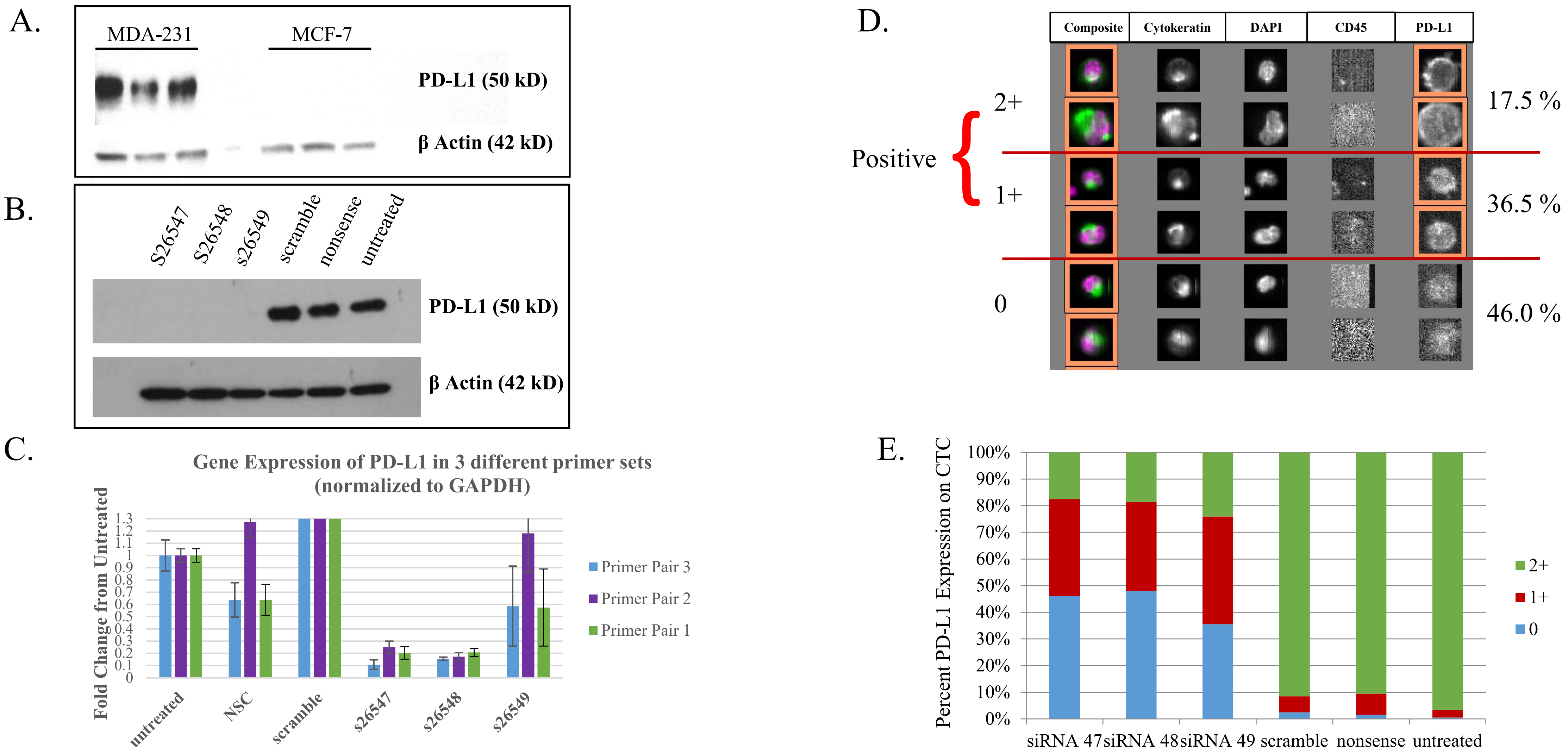

S6 Fig. PD-L1 sensitivity and specificity of antibody clone 29E.2A3 for PD-L1. A. Western blot of cell lysates from cultured human breast cancer cell lines known to express (MDA-MB-231) or not to express PD-L1 (MCF-7) using E1L3N Rabbit mAb, confirming the PD-L1 status of these two cell lines. B. PD-L1 protein expression by Western blot of lysates of MDA-MB-231 cell line (PD-L1 positive) treated with siRNA against PD-L1. PD-L1 expression was determine with E1L3N Rabbit mAb. Clones of MDA-MB-231 cells treated with siRNA against PD-L1 (s26547, s26548, and s26549) or with scrambled or nonsense probes, confirming the success of the PD-L1 knockdown process. C. PD-L1 gene expression analysis of MDA-MB-231 cell lines treated with siRNA against PD-L1. Clones of MDA-MB-231 cells treated with siRNA against PD-L1 as described in B above, confirming the success of the PD-L1 knock down process. (■) Primer Pair 3 PD-L1; (■) Primer Pair 2 PD-L1; (■) Primer Pair 1 PD-L1. D. Thumbnail gallery images of PD-L1 protein expression on MDA-MB-231 cell lines (wild type and PD-L1 siRNA treated cell lines when processed by CellSearch® (see details in B above). This illustration is from an MDA-MB-231 clone treated with siRNA against PD-L1 (siRNA s26547). The 5th column represents fluorescent staining for PD-L1. 0, 1+ and 2+ represent visual scoring for PD-L1 as described. E. PD-L1 protein expression when processed by CellSearch® on MDA-MB-231 cell lines (wild type and PD-L1 siRNA treated cell lines (see details in B above). Graphic representation of fluorescent staining for PD-L1 when visually scored as 0-2+ (see Supplementary Methods for details). Wild type MDA-MB-231 and clones treated with scrambled and nonsense probes have high percentage (≥90%) of 2+ fluorescent staining for PD-L1. Clones treated with siRNA (s26547, s26548, and s26549) have <25% 2+, and ≥75% staining with 0-1+. These data are concordant with B and C, and strongly suggest that clone 29E.2A3 is staining PD-L1 protein.
