# Supplementary material for: PD-L1 expression on circulating tumor cells and platelets in patients with metastatic breast cancer
Source: PLoS One. 2021 Nov 15;16(11):e0260124. doi: 10.1371/journal.pone.0260124 (PMC8592410; doi:10.1371/journal.pone.0260124)
Supplement: S7 Fig — A. MDA-MB-231: 97.5% of cells were strongly PD-L1 positive (2+), 2.5% of cells were PD-L1 negative (0). B. MDA-MB-468: 7.4% of cells were strongly PD-L1 positive (2+), 6.6% of cells were weakly PD-L1 positive (1+), and 86% of cells were PD-L1 negative (0). C. Sk-Br-3: 3% of cells were strongly PD-L1 positive (2+), 6% of cells were weakly PD-L1 positive (1+), and 91% of cells were PD-L1 negative (0). D. BT-474: 0.01% of cells were weakly PD-L1 positive (1+), and 99.99% of cells were PD-L1 negative (0). E. MCF-7: 100% of cells were PD-L1 negative (0). (PDF) [file pone.0260124.s008.pdf]

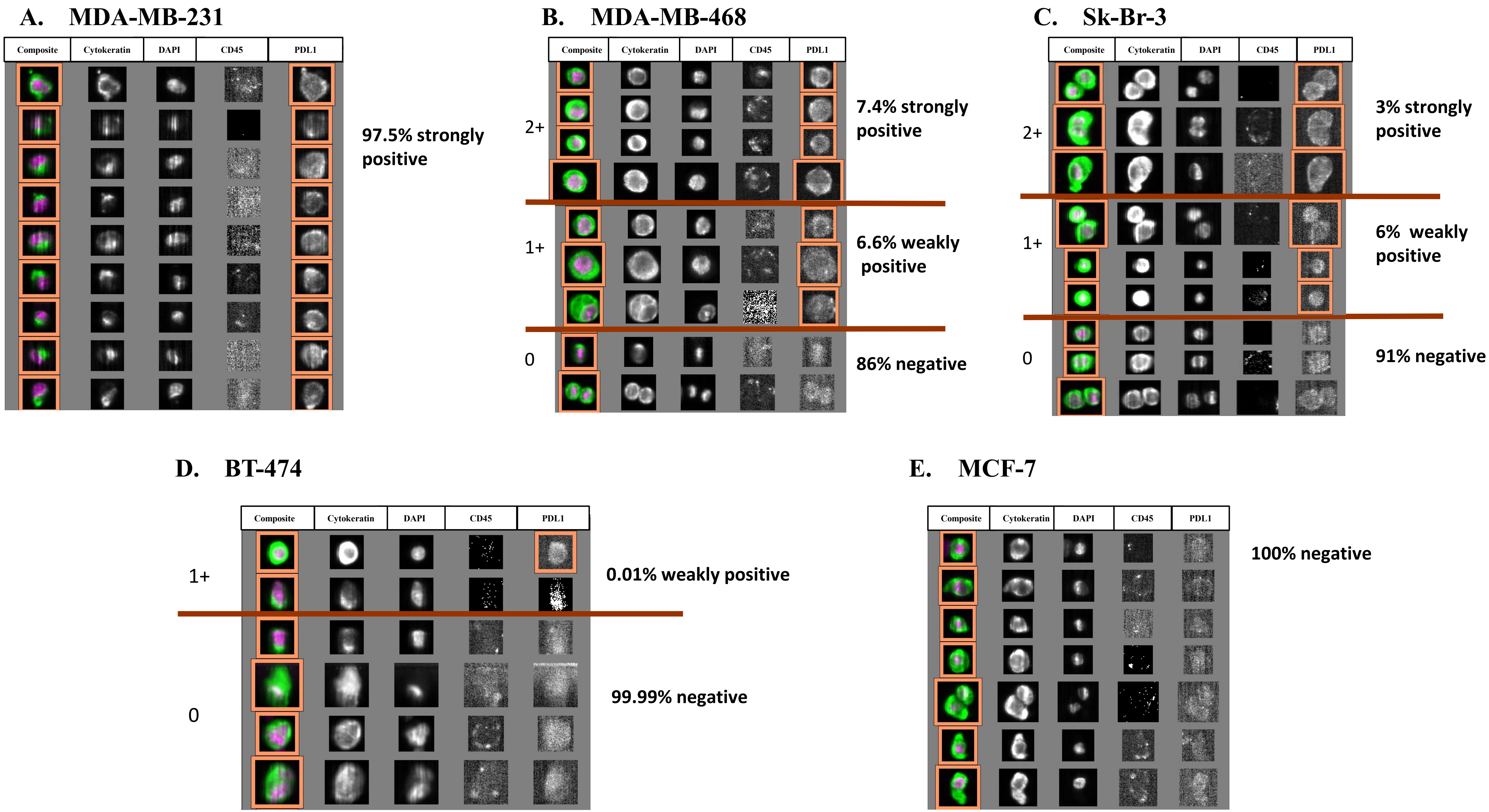

S7 Fig. PD-L1 expression on cultured breast cancer cell lines spiked into healthy donor whole blood and processed through CellSearch®. A. MDA-MB-231: 97.5% of cells were strongly PD-L1 positive (2+), 2.5% of cells were PD-L1 negative (0). B. MDA-MB-468: 7.4% of cells were strongly PD-L1 positive (2+), 6.6% of cells were weakly PD-L1 positive (1+), and 86% of cells were PD-L1 negative (0). C. Sk-Br-3: 3% of cells were strongly PD-L1 positive (2+), 6% of cells were weakly PD-L1 positive (1+), and 91% of cells were PD-L1 negative (0). D. BT-474: 0.01% of cells were weakly PD-L1 positive (1+), and 99.99% of cells were PD-L1 negative (0). E. MCF-7: 100% of cells were PD-L1 negative (0).
