# Supplementary material for: PD-L1 expression on circulating tumor cells and platelets in patients with metastatic breast cancer
Source: PLoS One. 2021 Nov 15;16(11):e0260124. doi: 10.1371/journal.pone.0260124 (PMC8592410; doi:10.1371/journal.pone.0260124)
Supplement: S8 Fig — A. CellSearch® thumbnail images, with each row representing a single cell and each column representing fluorescence of protein markers. The 5th column illustrates fluorescent staining for PD-L1. B. A single frame within a CellSearch® cartridge. Magnification of a single frame (highlighted with red), one of the 175 frames. C. Examples of CellSearch® frames with platelet PD-L1 staining count of >1,000, 100-1,000, <100, and 0. Samples with an average number of platelet PD-L1/3 frames of >1,000 and 100-1,000 PD-L1 were arbitrarily designated positive for PD-L1 staining. CellSearch® frames with 0 and <100 PD-L1 positive platelets per frame were designated as negative for PD-L1 staining. (PDF) [file pone.0260124.s009.pdf]

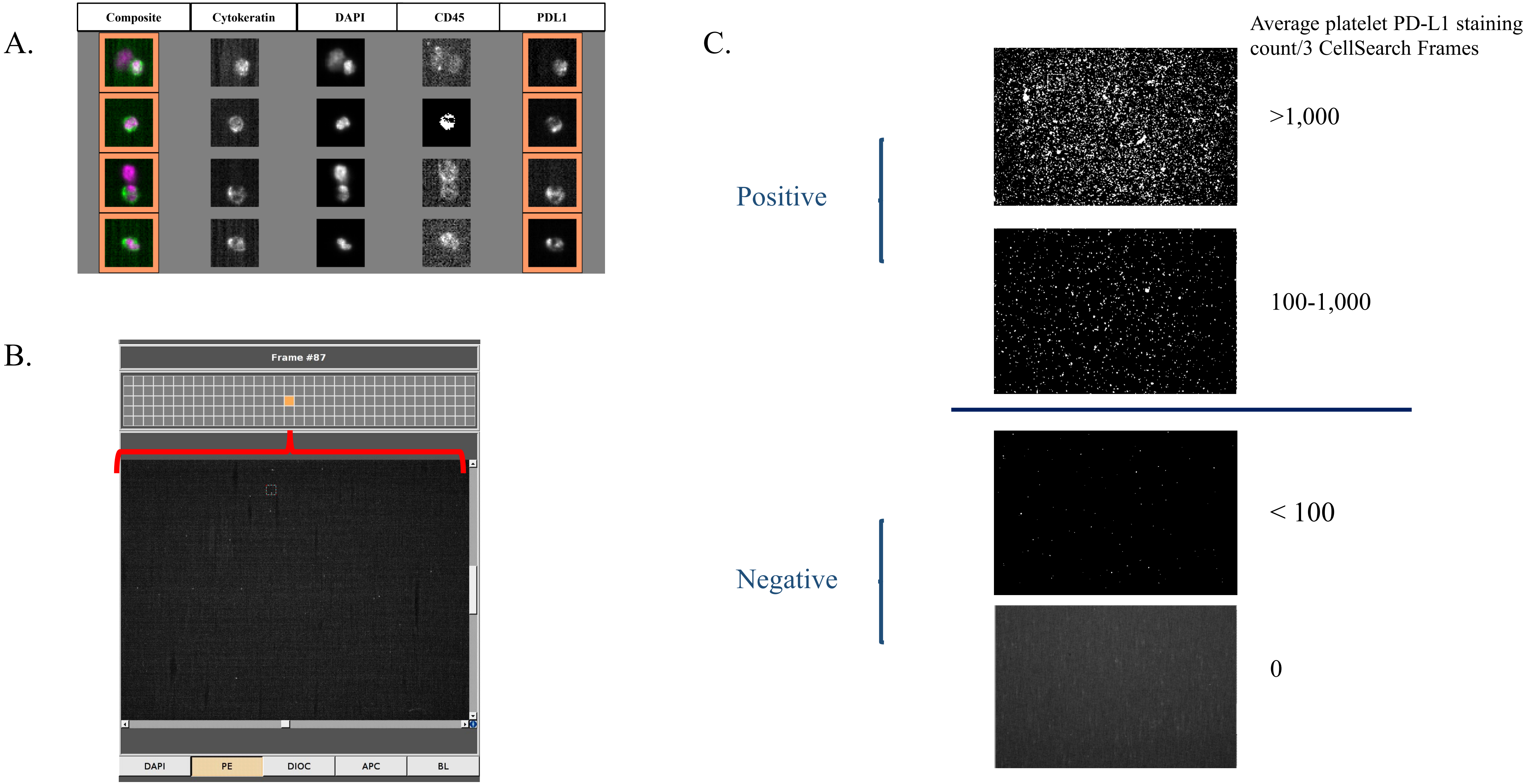

S8 Fig. Semi-quantitative scale of platelets PD-L1 positivity per CellSearch® frame. A. CellSearch® thumbnail images, with each row representing a single cell and each column representing fluorescence of protein markers. The 5th column illustrates fluorescent staining for PD-L1. B. A single frame within a CellSearch® cartridge. Magnification of a single frame (highlighted with red), one of the 175 frames. C. Examples of CellSearch® frames with platelet PD-L1 staining count of >1,000, 100-1,000, <100, and 0. Samples with an average number of platelet PD-L1/3 frames of >1,000 and 100-1,000 PD-L1 were arbitrarily designated positive for PD-L1 staining. CellSearch® frames with 0 and <100 PD-L1 positive platelets per frame were designated as negative for PD-L1 staining.
