# Supplementary material for: PD-L1 expression on circulating tumor cells and platelets in patients with metastatic breast cancer
Source: PLoS One. 2021 Nov 15;16(11):e0260124. doi: 10.1371/journal.pone.0260124 (PMC8592410; doi:10.1371/journal.pone.0260124)
Supplement: S1 Table — The characteristics for ER, PgR, HER2 expression as well as cell culture media information are provided. (PDF) [file pone.0260124.s010.pdf]

S1 Table. PD-L1 expression as determined by CellSearch® in cultured cell lines. The characteristics for ER, PgR, HER2 expression[a] as well as cell culture media information are provided.

Cell line	ER	PgR	HER-2	PD-L1 %[b]	Cell Culture Media
**MDA-MB-231**	-	-	-	97.5	EMEM + 10% FBS
**MDA-MB-468**	-	-	-	14.1	DMEM + 10% FBS
**Sk-Br-3**	-	-	+	9	DMEM + 10% FBS
**BT-474**	+	+	+	0.005	DMEM + 10% FBS
**MCF-7**	+	+	-	0	DMEM + 10% FBS

[a] ER, PgR, and HER2 positive or negative status according to previous cell culture work [3].

[b] PD-L1 % represents results from CellSearch® in vitro spike-in experiments.
