# Supplementary material for: PD-L1 expression on circulating tumor cells and platelets in patients with metastatic breast cancer
Source: PLoS One. 2021 Nov 15;16(11):e0260124. doi: 10.1371/journal.pone.0260124 (PMC8592410; doi:10.1371/journal.pone.0260124)
Supplement: S2 Table — (PDF) [file pone.0260124.s011.pdf]

S2 Table. Platelet PD-L1 expression in Fixed versus Non-Fixed Whole Blood

	**Platelet PD-L1 Expression (+/-)[a]**	
**Patient ID**	**CellSave (Fixed WB)**	**EDTA (Non-Fixed WB)**
1	+	+
2	-	-
3	+	+
4	+	+
5	+	+
6	+	+
7	-	-
8	-	+
9	+	+
10	+	+
11	+	+
12	-	-
13	+	+

[a] Considered positive (+) if ≥100 PD-L1 platelets/CellSearch® frame; considered negative (-) if 0- 99 PD-L1 platelets/CellSearch® frame; WB= whole blood.
