# Supplementary material for: PD-L1 expression on circulating tumor cells and platelets in patients with metastatic breast cancer
Source: PLoS One. 2021 Nov 15;16(11):e0260124. doi: 10.1371/journal.pone.0260124 (PMC8592410; doi:10.1371/journal.pone.0260124)
Supplement: S3 Table — (PDF) [file pone.0260124.s012.pdf]

S3 Table. Platelet PD-L1 expression according to platelet count in CellSearch® cartridges

Sample	Platelet Count (1,000/ul)		Platelet PD-L1 Staining [c]	Category [d]
	CBC [a]	Cartridge [b]		
1	425	2	<100	Negative
2	224	3	<100	
3	238	1	<100	
4	159	1	<100	
5	262	1	100-1,000	Positive
6	330	2	100-1,000	
7	383	2	>1,000	

[a] Platelet count determined in routine clinical blood count (CBC) drawn the same day as study specimen.

[b] Count of platelets carried over into the CellSearch cartridge after processing the original 7.5ml WB for CTC enrichment through CellSearch® assay (see Methods and Supplemental Material text for details).

[c] Average platelet count/3 CellSearch Frames (see Supplemental Material for details).

[d] Classification of platelet PD-L1 staining (see Supplemental Material text for details).
