# Supplementary material for: PD-L1 expression on circulating tumor cells and platelets in patients with metastatic breast cancer
Source: PLoS One. 2021 Nov 15;16(11):e0260124. doi: 10.1371/journal.pone.0260124 (PMC8592410; doi:10.1371/journal.pone.0260124)
Supplement: S4 Table — (PDF) [file pone.0260124.s013.pdf]

S4 Table. Patient demographic characteristics.

Characteristics	Category	Total (N=124)
**Breast Cancer Type**		**N (%)**
	Lobular	13 (10.5%)
	Ductal	97 (78.2%)
	Mixed Lobular and Ductal	9 (7.3%)
	Missing	5 (4.0%)
**Primary tumor hormone receptor status**		**N (%)**
	Triple Neg	14 (11.3%)
	ER+ HER2-	67 (54.0%)
	HER2+	24 (19.4%)
	Unknown	19 (15.3%)
**1st clinical met hormone receptor status**		
	Triple Neg	18 (14.5%)
	ER+ HER2-	70 (56.5%)
	HER2+	27 (21.8%)
	Unknown	9 (7.3%)
**Most recent metastatic hormone receptor status** [a]		
	Triple Neg	22 (17.7%)
	ER+ HER2-	70 (56.5%)
	HER2+	26 (21.0%)
	Unknown	6 (4.8%)
**Disease Site**	Bone only	11 (8.9%)
	Bone + other site	67 (54.0%)

	Other site (no bone)	46 (37.1%)
	Liver Present vs. Absent	63 (50.8%) vs 61 (49.2%)
**Therapy [b]**		**N (%)**
Chemotherapy		52 (41.9%)
Endocrine Therapy		45 (36.3%)
Anti-HER2 Therapy		21 (16.9%)
CDK4/6 inhibitor		15 (12.1%)
PARP inhibitor		3 (2.4%)
Bone modifying agent		55 (44.4%)
zoledronic acid		32 (25.8%)
denosumab		22 (17.7%)
**Anticoagulants [c]**		91 (73.4%)
rivaroxaban (anti-thrombin)		5 (4.0%)
enoxaparin (anti-Factor X)		5 (4.0%)
apixaban (anti-Factor X)		2 (1.6%)
clopidogrel (anti-platelet aggregation)		1 (0.8%)
**Drug Administration Route**		**N (%)**
Intravenous		48 (38.7%)
Intramuscular		16 (12.9%)
Oral		58 (46.8%)
**Blood tests**		
**CBC counts (Unit)**	**N (# pts missing)**	**Mean (SD)**
WBC (K/ul)	120 (4)	6.1 (3.49)
Platelet (K/ul)	120 (4)	242.7 (85.12)
RBC (M/ul)	120 (4)	3.9 (0.57)
Neutrophil (%)	115 (9)	64.7 (12.33)

Absolute Neutrophil	115 (9)	4.2 (3.25)
**Recent procedure**	Yes	30 (24.2%)
**Tissue Biopsy**		57 (46.0%)
**Intravascular port placement**		63 (50.8%)
**Smoking status**		**N (%)**
	Never/passive smoker	70 (56.5%)
	Former smoker	49 (39.5%)
	Current some or every smoker	5 (4.0%)
**Co-Morbid illness**		**N (%)**
Diabetes Status	Yes	15 (12.1%)

[a] The metastatic biopsy that was performed closest to the time the blood specimen for this study was collected

[b] Therapy variable illustrates either the therapy the patient was currently on or the last therapy on which the patient progressed at the time of 1st blood draw (Timepoint-1). Therapy categories were not mutually exclusive as some patients were on multiple therapies simultaneously.

[c] 78 patients were taking NSAIDs and/or acetaminophen on intermittent basis as-needed, and it was not documented in the patient chart whether they had taken the drug at the time of the 1st blood draw (timepoint-1).
