# Supplementary material for: PD-L1 expression on circulating tumor cells and platelets in patients with metastatic breast cancer
Source: PLoS One. 2021 Nov 15;16(11):e0260124. doi: 10.1371/journal.pone.0260124 (PMC8592410; doi:10.1371/journal.pone.0260124)
Supplement: S5 Table — (PDF) [file pone.0260124.s014.pdf]

S5 Table. Univariable association of factors of interest with CTC PD-L1 positive rate

**Characteristics**	**Category**	**Rate ratio (95% CI)** [a]	**P-value** [b]
**Breast Cancer Type**	(Overall)		0.032
	Ductal vs. Lobular	0.32 (0.136, 0.752)	0.009
	Mixed Lobular and Ductal vs. Lobular	0.51 (0.144, 1.783)	0.289
**Primary tumor**	(Overall)		0.095
	ER+, HER2- vs. Triple Neg	1.18 (0.179, 7.728)	0.866
	HER2+ vs. Triple Neg	3.49 (0.480, 25.428)	0.217
**1st clinical metastasis**	**(Overall)**		**<0.001**
	**ER+, HER2- vs. Triple Neg**	**0.33 (0.172, 0.616)**	**<0.001**
	**HER2+ vs. Triple Neg**	**0.22 (0.115, 0.431)**	**<0.001**
**Most recent metastasis** [c]	**(Overall)**		**<0.001**
	**ER+, HER2- vs. Triple Neg**	**0.31 (0.173, 0.564)**	**<0.001**
	**HER2+ vs. Triple Neg**	**0.22 (0.119, 0.401)**	**<0.001**
**Disease Site**	**(Overall)**		**<0.001**
	Bone + other site vs. Other site (no bone)	0.65 (0.219, 1.913)	0.432
	**Bone only vs. Other site (no bone)**	**0.14 (0.054, 0.335)**	**<0.001**
Liver metastases	Present vs. Absent	0.53(0.179, 1.552)	0.245
**Therapy** [d]			
Chemotherapy	Yes vs. No	0.31 (0.149, 0.651)	0.002
**Endocrine Therapy**	**Yes vs. No**	**3.19 (1.612, 6.310)**	**<0.001**
Anit-HER2 Therapy	Yes vs. No	0.51 (0.172, 1.487)	0.215
**CDK4/6 inhibitor**	**Yes vs. No**	**4.11 (2.464, 6.847)**	**<0.001**
PARP inhibitor	Yes vs. No	3.87 (0.443, 33.733)	0.221
Bone agent	Yes vs. No	1.91 (0.658, 5.573)	0.233

zoledronic Acid	Yes vs. No	1.79 (0.690, 4.622)	0.232
denosumab	Yes vs. No	0.73 (0.272, 1.982)	0.541
**Anticoagulants** [e]			
rivaroxaban	Yes vs. No	1.55 (0.442, 5.442)	0.494
enoxaparin	Yes vs. No	0.27 (0.067, 1.097)	0.067
apixaban	Yes vs. No	0.64 (0.212, 1.960)	0.439
**Drug Administration Route**			
Intravenous	Yes vs. No	0.37 (0.168, 0.829)	0.016
Intramuscular	Yes vs. No	1.26 (0.349, 4.521)	0.727
Oral	Yes vs. No	2.73 (1.268, 5.884)	0.01
**Blood Tests: CBC Counts**			
WBC (K/ul)	Continuous variable	0.92 (0.801, 1.054)	0.228
Platelet (K/ul)	Continuous variable	1.00 (1.000, 1.006)	0.024
RBC (M/ul)	Continuous variable	0.91 (0.522, 1.586)	0.738
Neutrophil (%)	Continuous variable	1.02 (1.001, 1.049)	0.039
Absolute Neutrophil	Continuous variable	0.97 (0.885, 1.064)	0.524
**Recent Procedure**			
Any	Yes vs. No	0.67 (0.223, 2.015)	0.477
Recent biopsy	Yes vs. No	0.42 (0.181, 0.995)	0.049
Intravenous Port Placement	Yes vs. No	1.00 (0.414, 2.424)	0.997
**Smoking status**	(Overall)		0.595
	Current some or every smoker vs. Never/passive smoker	3.07 (0.326, 28.862)	0.327
	Former smoker vs. Never/passive smoker	1.21 (0.427, 3.416)	0.723
**Comorbid Illness**			
Diabetes	Yes vs. No	4.05 (1.273, 12.881)	0.018

[a] CTC PD-L1 positivity was treated as the number of positive CTC and therefore described as a rate ratio.

[b] Statistical significance was considered for $p<0.001$.

[c] Most recent metastases and 1st clinical metastases are not mutually exclusive. The most recent metastases refers to the metastatic biopsy that was performed closest to the time the blood specimen for this study was collected

[d] Therapy variable illustrates either the therapy the patient was currently on or the last therapy the patient progressed on at the time of 1st blood draw. Therapy categories were not mutually exclusive as some patients were on multiple therapies simultaneously.

[e] Clopidogrel not included in analysis since only a single patient was taking it.
