# Supplementary material for: PD-L1 expression on circulating tumor cells and platelets in patients with metastatic breast cancer
Source: PLoS One. 2021 Nov 15;16(11):e0260124. doi: 10.1371/journal.pone.0260124 (PMC8592410; doi:10.1371/journal.pone.0260124)
Supplement: S6 Table — (PDF) [file pone.0260124.s015.pdf]

S6 Table. Multivariable results of factors of interest with CTC PD-L1 positivity rate

**Characteristics (unit)**	**Category**	**Rate ratio [a] (95% CI)**	**P-value [b]**
**Breast Cancer Type**	(Overall)		0.037
	Ductal vs. Lobular	0.51 (0.243, 1.072)	0.076
	Mixed Lobular and Ductal vs. Lobular	1.53 (0.733, 3.183)	0.258
**Most recent metastatic hormone receptor status [c]**	(Overall)		0.025
	**ER+, HER2- vs. Triple Neg**	**2.56 (1.298, 5.058)**	**0.007**
	**HER2+ vs. Triple Neg**	**3.14 (1.040, 9.492)**	**0.042**
**Disease Site**	**(Overall)**		**<0.001**
	**Bone + other site vs. Other site (no bone)**	**0.19 (0.078, 0.462)**	**<0.001**
	**Bone only vs. Other site (no bone)**	**0.09 (0.034, 0.243)**	**<0.001**
Chemotherapy	Yes vs. No	0.48 (0.216, 1.076)	0.075
**CDK4/6 inhibitor**	**Yes vs. No**	**3.60 (1.403, 9.238)**	**0.008**

[a] Rate ratio is calculated using Poisson GEE model assuming an independent correlation structure to explore the association between Platelet PD-L1 and factors of interest.

[b] Statistical significance is any $p<0.05$.

[c] The metastatic biopsy that was performed closest to the time the blood specimen for this study was collected.
