# Supplementary material for: PD-L1 expression on circulating tumor cells and platelets in patients with metastatic breast cancer
Source: PLoS One. 2021 Nov 15;16(11):e0260124. doi: 10.1371/journal.pone.0260124 (PMC8592410; doi:10.1371/journal.pone.0260124)
Supplement: S7 Table — (PDF) [file pone.0260124.s016.pdf]

S7 Table. Univariable association of factors of interest with Platelet PD-L1 positivity

**Characteristics**	**Category**	**Odds ratio [a] (95% CI)**	**P-value [b]**
**Breast Cancer Type**	(Overall)		0.042
	Ductal vs. Lobular	0.73 (0.565, 0.953)	0.02
	Mixed Lobular and Ductal vs. Lobular	0.88 (0.600, 1.286)	0.506
**Primary tumor**	(Overall)		0.002
	ER+, HER2- vs. Triple Neg	1.00 (0.761, 1.327)	0.973
	HER2+ vs. Triple Neg	0.78 (0.599, 1.022)	0.072
**1st clinical metastasis:**	(Overall)		0.047
	ER+, HER2- vs. Triple Neg	1.11 (0.887, 1.392)	0.36
	HER2+ vs. Triple Neg	0.91 (0.725, 1.141)	0.411
**Most recent metastasis [c]:**	(Overall)		0.025
	ER+, HER2- vs. Triple Neg	1.17 (0.957, 1.425)	0.126
	HER2+ vs. Triple Neg	0.94 (0.770, 1.149)	0.547
**Disease Site**	(Overall)		0.701
	Bone + other site vs. Other site (no bone)	1.06 (0.906, 1.244)	0.459
	Bone only vs. Other site (no bone)	1.09 (0.852, 1.397)	0.489
Liver Mets	Present vs. Absent	1.16 (1.005, 1.337)	0.043
**Therapy [d]**			
Chemotherapy	Yes vs. No	1.13 (0.996, 1.291)	0.057
Endocrine Therapy	Yes vs. No	0.94 (0.816, 1.084)	0.395
Anti-HER2 therapy	Yes vs. No	0.83 (0.715, 0.968)	0.017
CDK4/6 inhibitor	Yes vs. No	1.07 (0.855, 1.329)	0.569
PARP inhibitor	Yes vs. No	0.92 (0.603, 1.412)	0.712
Bone agent	Yes vs. No	1.06 (0.911, 1.225)	0.471

zoledronic Acid	Yes vs. No	1.17 (0.980, 1.385)	0.084
denosumab	Yes vs. No	0.89 (0.762, 1.048)	0.168
**Anticoagulants** [e]			
rivaroxaban	Yes vs. No	0.85 (0.655, 1.093)	0.202
enoxaparin	Yes vs. No	0.86 (0.657, 1.117)	0.253
apixaban	Yes vs. No	1.19 (0.835, 1.700)	0.335
**Drug Administration Route**			
Intravenous	Yes vs. No	1.02 (0.890, 1.159)	0.816
Intramuscular	Yes vs. No	0.81 (0.682, 0.953)	0.012
Oral	Yes vs. No	0.98 (0.858, 1.125)	0.795
**Blood Tests: CBC Counts (Unit)**			
WBC (K/ul)	Continuous variable	1.00 (0.977, 1.028)	0.877
Platelet (K/ul)	Continuous variable	1.00 (0.999, 1.001)	0.619
**RBC (M/ul)**	**Continuous variable**	**0.72 (0.637, 0.806)**	**<0.001**
Neutrophil (%)	Continuous variable	1.00 (0.993, 1.007)	0.953
Absolute Neutrophil	Continuous variable	1.00 (0.978, 1.033)	0.723
**CTC (100 CTC/7.5ml WB)**	**Continuous variable**	**1.03 (1.014, 1.050)**	**<0.001**
**≥5 CTC vs. <5 CTC**	**Yes vs. No**	**1.45 (1.28, 1.65)**	**<0.001**
**Recent procedure**			
Any	Yes vs. No	1.09 (0.928, 1.276)	0.3
Biopsy	Yes vs. No	0.96 (0.842, 1.092)	0.529
Intravenous port placement	Yes vs. No	1.04 (0.893, 1.222)	0.587
**Smoking status**	**(Overall)**		**<0.001**
	**Current some or every smoker vs. Never/passive smoker**	**0.76 (0.682, 0.836)**	**<0.001**
	Former smoker vs. Never/passive smoker	1.15 (0.981, 1.359)	0.083

**Co-Morbid illness**			
Diabetes	Yes vs. No	0.88 (0.717, 1.075)	0.208

[a] Platelet PD-L1 positivity is based on binary positive (≥100 PD-L1 positive platelets) or negative (<100 PD-L1 positive platelets), odds ratio is positive vs. negative PD-L1 expression on platelets compared to variables of interest.

[b] Statistical significance is any p<0.001.

[c] The metastatic biopsy that was performed closest to the time the blood specimen for this study was collected

[d] Therapy variable illustrates either the therapy the patient was currently on or the last therapy the patient progressed on at the time of 1st blood draw. Therapy categories were not mutually exclusive as some patients were on multiple therapies simultaneously.

[e] Clopidogrel not included in analysis since only a single patient was taking it.
