# Supplementary material for: PD-L1 expression on circulating tumor cells and platelets in patients with metastatic breast cancer
Source: PLoS One. 2021 Nov 15;16(11):e0260124. doi: 10.1371/journal.pone.0260124 (PMC8592410; doi:10.1371/journal.pone.0260124)
Supplement: S8 Table — (PDF) [file pone.0260124.s017.pdf]

S8 Table. Multivariable results of factors of interest with Platelet PD-L1 positivity

**Characteristics (unit)**	**Category**	**Odds ratio (95% CI)** [a]	**P-value** [b]
**RBC (M/ul)**	**Continuous variable**	**0.73 (0.642, 0.820)**	**<0.001**
Smoking status	(Overall)		0.017
	Current some or every smoker vs. Never/passive smoker	0.89 (0.773, 1.014)	0.078
	Former smoker vs. Never/passive smoker	1.14 (0.977, 1.320)	0.099

[a] Platelet PD-L1 positivity is binary positive (≥100 PD-L1 positive platelets) or negative (<100 PD-L1 positive platelets) by CellSearch®, odds ratio is calculated using GEE model assuming an independent correlation structure to explore the association between Platelet PD-L1 and factors of interest.

[b] Statistical significance is considered for any $p<0.05$.
