# Supplementary material for: PD-L1 expression on circulating tumor cells and platelets in patients with metastatic breast cancer
Source: PLoS One. 2021 Nov 15;16(11):e0260124. doi: 10.1371/journal.pone.0260124 (PMC8592410; doi:10.1371/journal.pone.0260124)
Supplement: S9 Table — (PDF) [file pone.0260124.s018.pdf]

S9 Table. Serial timepoints CTC, Platelet PDL1, treatment, days between serial timepoints

Patient ID	Timepoint 1 CTC Count (% PDL1+)	Timepoint 1 Platelet PDL1 level	Timepoint 1 Most Recent Therapy	Timepoint 1 OT/PD*	Days from Timepoint 1 to Timepoint 2	Timepoint 2 CTC Count (% PDL1+)	Timepoint 2 Platelet PDL1 level	Timepoint 2 Most Recent Therapy	Timepoint 2 OT/PD	Days from Timepoint 2 to Timepoint 3	Timepoint 3 CTC Count (% PDL1+)	Timepoint 3 Platelet PDL1 level	Timepoint 3 Most Recent Therapy	Timepoint 3 OT/PD	Days from Timepoint 3 to Timepoint 4	Timepoint 4 CTC Count (% PDL1+)	Timepoint 4 Platelet PDL1 level	Timepoint 4 Most Recent Therapy	Timepoint 4 OT/PD	Days from Timepoint 4 to Timepoint 5	Timepoint 5 CTC Count (% PDL1+)	Timepoint 5 Platelet PDL1 level	Timepoint 5 Most Recent Therapy	Timepoint 5 OT/PD
5	179 (0%)	>1000	Anastrozole	PD	490	109 (11.93%)	>1000	Capecitabine	OT	64	475 (4.67%)	>1000	Vinorelbine	OT	43	1731 (4.00%)	>1000	Vinorelbine	PD	230	102 (5.88%)	>1000	Eribulin	OT
6	0 (N/A)	0	Letrozole + Palbociclib	PD	287	5 (40.00%)	0	Exemestane + Everolimus	PD	252	41 (7.32%)	0	Doxorubicin	PD	--	--		--	--	--	--		--	--
16	1 (0%)	100-1000	Letrozole + Palbociclib	OT	343	53 (0%)	100-1000	Capecitabine	PD	63	159 (0%)	100-1000	Paclitaxel	OT	89	571 (0.33%)	>1000	Paclitaxel	PD	--	--		--	--
17	0 (N/A)	0	Eribulin + Trastuzumab	PD	70	1 (0%)	0	CMF + Trastuzumab	OT	34	0 (N/A)	0	CMF + Trastuzumab	PD	--	--		--	--	--	--		--	--
26	594 (0.84%)	0	Anastrozole	PD	223	70 (0%)	0	Paclitaxel	PD	109	28 (3.57%)	0	Capecitabine	OT	71	1091 (0.67%)	<100	Liposomal Doxorubicin	OT	147	1864 (1.00%)	>1000	Eribulin	PD
31	4 (0%)	<100	Capecitabine	PD	9	7 (0%)	<100	Capecitabine	PD	--	--		--	--	--	--		--	--	--	--		--	--
35	0 (N/A)	0	Fulvestrant	PD	105	10 (0%)	0	Capecitabine	PD	--	--		--	--	--	--		--	--	--	--		--	--
37	11 (0%)	100-1000	Capecitabine	PD	14	3 (0%)	<100	Capecitabine	PD	124	62 (3.23%)	100-1000	Vinorelbine	PD	--	--		--	--	--	--		--	--
43	8 (0%)	>1000	Gemcitabine	PD	111	5 (20.00%)	>1000	Doxorubicin	PD	--	--		--	--	--	--		--	--	--	--		--	--
45	88 (1.14%)	<100	CMF	PD	140	21 (0%)	100-1000	Eribulin	PD	--	--		--	--	--	--		--	--	--	--		--	--
46	3 (0%)	<100	Carboplatin + Trastuzumab	PD	392	0 (N/A)	0	Gemcitabine + Trastuzumab	PD	--	--		--	--	--	--		--	--	--	--		--	--
48	14 (0%)	0	Vinorelbine	PD	112	125 (6.40%)	<100	Doxorubicin	PD	--	--		--	--	--	--		--	--	--	--		--	--
50	0 (N/A)	0	Capecitabine	PD	76	2 (0%)	0	Tamoxifen + Trastuzumab	OT	21	3 (0%)	0	Tamoxifen + Trastuzumab	PD	--	--		--	--	--	--		--	--
53	26 (0%)	<100	None	PD	133	0 (N/A)	100-1000	Paclitaxel + Trastuzumab + Pertuzumab	OT	--	--		--	--	--	--		--	--	--	--		--	--
55	2 (0%)	>1000	None	PD	154	69 (2.90%)	>1000	Gemcitabine	PD	--	--		--	--	--	--		--	--	--	--		--	--
56	0 (N/A)	0	Anastrozole	PD	87	9 (0%)	<100	Fulvestrant + Palbociclib	PD	--	--		--	--	--	--		--	--	--	--		--	--
57	4 (25.00%)	<100	Carboplatin + Vinorelbine + Trastuzumab	PD	30	1 (100.00%)	<100	Tocilizumab + Trastuzumab	PD	--	--		--	--	--	--		--	--	--	--		--	--
58	0 (N/A)	<100	None	PD	189	0 (N/A)	100-1000	Paclitaxel +/- Reparaxin	PD	--	--		--	--	--	--		--	--	--	--		--	--
61	10 (0%)	<100	Fulvestrant	PD	400	5 (0%)	100-1000	Capecitabine	PD	--	--		--	--	--	--		--	--	--	--		--	--
62	3 (0%)	0	Letrozole + Palbociclib	PD	373	0 (N/A)	<100	Capecitabine	PD	86	4 (0%)	<100	Fulvestrant + Everolimus	PD	--	--		--	--	--	--		--	--
65	0 (N/A)	<100	Trastuzumab + Pertuzumab	PD	168	0 (N/A)	0	Trastuzumab + Pertuzumab	PD	42	0 (N/A)	0	Vinorelbine + Trastuzumab + Pertuzumab	OT	70	0 (N/A)	100-1000	Trastuzumab + Pertuzumab	OT	--	--		--	--
66	4 (0%)	>1000	Capecitabine	PD	27	91 (0%)	>1000	Olaparib	PD	--	--		--	--	--	--		--	--	--	--		--	--

**68**	227 (1.32%)<br>0	Paclitaxel +/- Reparixin	OT	371	4038 (9.00%)<br>>1000	Letrozole + Palbociclib	PD	--	--	--	--	--	--	--	--	--	--	--	--
**70**	0 (N/A)<br>100-1000	Cyclophosphamide	OT	63	4 (25.00%)<br><100	Cyclophosphamide	OT	--	--	--	--	--	--	--	--	--	--	--	--
**71**	0 (N/A)<br>0	Capecitabine	OT	49	0 (N/A)<br>0	Capecitabine	PD	--	--	--	--	--	--	--	--	--	--	--	--
**73**	7 (0%)<br><100	Doxorubicin	OT	58	11 (9.09%)<br><100	None	PD	--	--	--	--	--	--	--	--	--	--	--	--
**74**	7 (0%)<br><100	WBRT	PD	84	25 (0%)<br>100-1000	Cisplatin +/- Velaparib	PD	--	--	--	--	--	--	--	--	--	--	--	--
**75**	0 (N/A)<br>0	None	PD	54	0 (N/A)<br>0	None	PD	--	--	--	--	--	--	--	--	--	--	--	--
**76**	2 (0%)<br><100	Exemestane + Everolimus	PD	116	1 (0%)<br><100	Fulvestrant + Palbociclib	PD	--	--	--	--	--	--	--	--	--	--	--	--
**77**	0 (N/A)<br><100	nab-Paclitaxel	PD	56	1 (100.00%)<br>0	Letrozole + Palbociclib	OT	--	--	--	--	--	--	--	--	--	--	--	--
**79**	19 (0%)<br>0	Doxorubicin	PD	57	10 (0%)<br><100	Tamoxifen	OT	--	--	--	--	--	--	--	--	--	--	--	--
**81**	2 (0%)<br>0	Paclitaxel	OT	84	2 (0%)<br><100	Paclitaxel	OT	210	12 (8.33%)<br>>1000	Letrozole + Palbociclib	PD	35	0 (N/A)<br>100-1000	Capecitabine	OT	--	--	--	--
**82**	0 (N/A)<br><100	Paclitaxel	PD	70	2 (100.00%)<br>0	Letrozole + Palbociclib	OT	--	--	--	--	--	--	--	--	--	--	--	--
**83**	21 (0%)<br>0	Vinorelbine	OT	77	48 (0%)<br><100	Vinorelbine	OT	21	36 (0%)<br>100-1000	Vinorelbine	PD	--	--	--	--	--	--	--	--
**84**	11 (0%)<br>100-1000	Capecitabine	OT	51	639 (0%)<br>>1000	Capecitabine	PD	--	--	--	--	--	--	--	--	--	--	--	--
**85**	1 (100.00%)<br>0	Letrozole	PD	182	0 (N/A)<br>100-1000	RAD-140	PD	--	--	--	--	--	--	--	--	--	--	--	--
**86**	0 (N/A)<br>0	Tamoxifen	PD	70	0 (N/A)<br>0	Paclitaxel + Trastuzumab + Pertuzumab	OT	--	--	--	--	--	--	--	--	--	--	--	--
**87**	2 (0%)<br>0	Letrozole + Palbociclib	PD	109	16 (6.25%)<br>>1000	Vinorelbine	PD	--	--	--	--	--	--	--	--	--	--	--	--
**88**	5 (0%)<br>0	Doxorubicin	PD	64	3 (33.33%)<br>0	Eribulin	OT	112	2 (0%)<br><100	RT	PD	--	--	--	--	--	--	--	--
**90**	0 (N/A)<br>100-1000	Letrozole + Palbociclib	OT	62	9 (100%)<br>100-1000	Letrozole + Palbociclib	OT	--	--	--	--	--	--	--	--	--	--	--	--
**91**	7 (0%)<br>>1000	Anastrozole + Palbociclib	OT	63	16 (0%)<br>100-1000	Anastrozole + Palbociclib	PD	--	--	--	--	--	--	--	--	--	--	--	--
**92**	122 (7.38%)<br>0	Fulvestrant	OT	49	60 (8.33%)<br>100-1000	Eribulin	OT	77	352 (8.81%)<br>100-1000	Gemcitabine	PD	--	--	--	--	--	--	--	--
**93**	0 (N/A)<br>0	Tamoxifen	PD	105	0 (N/A)<br>0	nab-Paclitaxel + Trastuzumab + Pertuzumab	OT	--	--	--	--	--	--	--	--	--	--	--	--
**96**	0 (N/A)<br><100	Fulvestrant	PD	56	0 (N/A)<br><100	Doxorubicin	OT	--	--	--	--	--	--	--	--	--	--	--	--
**97**	27 (0%)<br>100-1000	Carboplatin	OT	30	17 (0%)<br>>1000	Eribulin	OT	--	--	--	--	--	--	--	--	--	--	--	--
**98**	4 (0%)<br>0	None	PD	84	2 (50.00%)<br>0	Doxorubicin	OT	37	2 (50.00%)<br><100	Doxorubicin	OT	47	3 (0%)<br><100	Doxorubicin	PD	--	--	--	--
**99**	12 (100.00%)<br>0	Olaparib	OT	156	21 (9.52%)<br><100	None	PD	--	--	--	--	--	--	--	--	--	--	--	--

**100**	0 (N/A)<br><100	Fulvestrant + Palbociclib	OT	55	0 (N/A)<br><100	Fulvestrant + Palbociclib	OT	--	--	--	--	--	--	--	--	--	--	--	--
**101**	31 (3.23%)<br><100	None	PD	66	71 (0%)<br><100	Vinorelbine	OT	--	--	--	--	--	--	--	--	--	--	--	--
**103**	6 (0%)<br>0	Trastuzumab + Pertuzumab	PD	84	0 (N/A)<br><100	ado-Trastuzumab Emtansine	OT	84	9 (11.11%)<br>100-1000	ado-Trastuzumab Emtansine	PD	--	--	--	--	--	--	--	--
**105**	3 (66.67%)<br><100	None	PD	56	2 (0%)<br><100	Capecitabine	OT	65	0 (N/A)<br><100	Eribulin	PD	--	--	--	--	--	--	--	--
**106**	0 (N/A)<br><100	ado-Trastuzumab Emtansine	OT	70	0 (N/A)<br><100	ado-Trastuzumab Emtansine	OT	--	--	--	--	--	--	--	--	--	--	--	--
**107**	0 (N/A)<br>0	None	PD	57	0 (N/A)<br>0	Capecitabine	PD	--	--	--	--	--	--	--	--	--	--	--	--
**109**	50 (2.00%)<br><100	Anastrozole	PD	56	50 (0%)<br><100	Fulvestrant	OT	--	--	--	--	--	--	--	--	--	--	--	--
**110**	0 (N/A)<br>0	Letrozole	OT	23	0 (N/A)<br><100	Letrozole + Palbociclib	PD	--	--	--	--	--	--	--	--	--	--	--	--
**111**	1 (0%)<br>0	Capecitabine	PD	84	11 (0%)<br><100	Fulvestrant + Palbociclib	PD	--	--	--	--	--	--	--	--	--	--	--	--
**112**	1 (0%)<br>100-1000	Paclitaxel + Trastuzumab + Pertuzumab	OT	63	0 (N/A)<br><100	Paclitaxel + Trastuzumab + Pertuzumab	OT	--	--	--	--	--	--	--	--	--	--	--	--
**114**	0 (N/A)<br><100	Gemcitabine + Trastuzumab + Pertuzumab	OT	70	0 (N/A)<br>100-1000	Gemcitabine + Trastuzumab + Pertuzumab	OT	--	--	--	--	--	--	--	--	--	--	--	--
**117**	556 (1.00%)<br>100-1000	Letrozole + Neratinib + IT Trastuzumab	PD	63	0 (N/A)<br>100-1000	ado-Trastuzumab Emtansine + IT Trastuzumab	OT	--	--	--	--	--	--	--	--	--	--	--	--

***OT= On Treatment, PD= Progressing Disease**
-- = blood was not collected at this timepoint
Abbreviations: CMF= Cyclophosphamide Methotrexate Fluorouracil; IT= Intrathecal; RT= Radiation Therapy; WBRT= Whole Brain Radiation Therapy
